# Dynamics of a geminivirus-encoded pre-coat protein and host RNA-dependent RNA polymerase 1 in regulating symptom recovery in tobacco

**DOI:** 10.1093/jxb/ery043

**Published:** 2018-02-08

**Authors:** Saumik Basu, Nirbhay Kumar Kushwaha, Ashish Kumar Singh, Pranav Pankaj Sahu, R Vinoth Kumar, Supriya Chakraborty

**Affiliations:** Molecular Virology Laboratory, School of Life Sciences, Jawaharlal Nehru University, New Delhi, India

**Keywords:** *Begomovirus*, *Geminiviridae*, pre-coat protein, RNA-dependent RNA polymerase 1, RNA silencing, short interfering RNA, symptom remission

## Abstract

RNA silencing is an integral part of the cellular defense mechanisms in plants that act against virus infection. However, the specific role of RNA silencing and the interplay between host and virus components during recovery from geminivirus infection remains unknown. Hence, in this study we aimed to examine the mechanism behind the host-specific recovery of *Nicotiana tabacum* infected with *Tomato leaf curl Gujarat virus* (ToLCGV). Unlike *Tomato leaf curl New Delhi virus* (ToLCNDV), ToLCGV infection resulted in symptom remission in *N. tabacum*, and we found that this was mainly due to cross-talk between the pre-coat protein (encoded by the AV2 ORF) of the virus and the host RNA-silencing component RNA-dependent RNA polymerase 1 (encoded by *NtRDR1*) of *N. tabacum*. Moreover, apart from the AV2 mutant, other mutants of ToLCNDV developed severe symptoms on a transgenic *NtRDR1*-overexpression line of *N. benthamiana*. In contrast, inoculation with ToLCGV resulted in symptom remission, which was due to enhanced methylation of the ToLCGV promoter. Our study reveals a novel ‘arms race’ in which the pre-coat protein of ToLCNDV selectively blocks the recovery process through inhibiting host-specific RDR1-mediated antiviral silencing in tobacco.

## Teaser

ToLCNDV-AV2 is essential for repression of - antiviral silencing mediated by RNA-dependent RNA polymerase 1 and is responsible for contrasting symptom development in *Nicotiana* species infected with different geminiviruses.

## Introduction

Tomato, an economically important vegetable crop, is continuously under threat of geminivirus infection, which leads to severe yield losses worldwide (Padidam *et al.*, 1995; Chakraborty, 2008; Hanssen *et al.*, 2010). *Tomato leaf curl New Delhi virus* (ToLCNDV) and *Tomato leaf curl Gujarat virus* (ToLCGV) are the predominant begomovirus species within the *Geminiviridae* family that cause severe leaf curl diseases of tomato and other vegetable crops in India (Chakraborty, 2008; Ranjan *et al.*, 2013; Kumar *et al.*, 2015). Their genomes share 74% similarity, although they have been classified as distinct species. ToLCNDV possesses a typical bipartite genome consisting of DNA-A and DNA-B encapsidated within two separate virions (Padidam *et al.*, 1995). Although ToLCGV is a monopartite begomovirus, association with DNA-B increases symptom severity (Chakraborty *et al.*, 2008; Ranjan *et al.*, 2013). The DNA-B component of ToLCNDV is indispensable for systemic infection in tomato plants (Padidam *et al.*, 1995; Chakraborty, 2008; Ranjan *et al.*, 2014). DNA-A mostly codes for proteins associated with replication and transcriptional activation, while DNA-B encodes proteins that facilitate intra- and intercellular movement (Hanley-Bowdoin *et al.*, 2013; Kushwaha *et al.*, 2017). Among the various proteins encoded in their genomes, the pre-coat protein (encoded by the AV2 ORF) shares 78% identity between ToLCGV and ToLCNDV.

Plants naturally defend themselves against invading viruses by transcriptional gene silencing (TGS) and post-transcriptional gene silencing (PTGS), and viruses have evolved to encode suppressor proteins to counteract these host defenses (Pumplin and Voinnet, 2013). For example, the TrAP protein that is encoded by *AC2* functions as a suppressor of TGS (Sharma and Ikegami, 2010; Amin *et al.*, 2011; Luna *et al.*, 2012). Similarly, the *AC4*/*C4*-encoded protein is responsible for both pathogenesis and suppression of silencing (Vanitharani *et al.*, 2004; Chellappan *et al.*, 2005; Kon *et al.*, 2007; Amin *et al.*, 2011). The *V2*-encoded pre-coat protein has been demonstrated to be a suppressor of both TGS and PTGS (Sharma and Ikegami, 2010; Wang *et al.*, 2014*a*).

Virus-infected plants can occasionally overcome infection through a process termed recovery/remission. Long double-stranded RNAs (dsRNAs), generated by bi-directional transcription of the geminivirus genome, sequentially induce RNA silencing in plants (Chellappan *et al.*, 2004; Akbar *et al.*, 2012; Hanley-Bowdoin *et al.*, 2013; Ashraf *et al.*, 2014). During RNA silencing, dsRNAs are cleaved by Dicer-like (DCL) enzymes into short interfering RNAs (siRNAs) of 21–24 nucleotide (nt) length to carry out either sequence-specific degradation of complementary mRNA or methylation of the viral promoter (Aregger *et al.*, 2012; Pumplin and Voinnet, 2013; Matzke and Matzke, 2014; Sahu *et al.*, 2014). RNA-dependent RNA polymerase 1 (RDR1) has been shown to play a significant role in antiviral defense and/or enhanced resistance in *Arabidopsis thaliana*, *Nicotiana benthamiana*, *N. tabacum*, and *Medicago truncatula* (Yu *et al.*, 2003; Yang *et al.*, 2004; Ying *et al.*, 2010). Differential expression of RDR1 has been shown to provide basal as well as induced resistance against *Tobacco mosaic virus* (TMV), and altered expression levels have been observed during exogenous application of the defense-associated phytohormone salicylic acid (SA) (Lee *et al.*, 2016). This suggests that RDR1 could be hormonally regulated during virus infection.

Although both RDR1 and RDR6 are encoded within the *N. tabacum* genome, only *RDR1* has been functionally characterized as a defense factor, acting against TMV, *Potato virus X* (PVX), and *Potato virus Y* (PVY) (Xie *et al.*, 2001; Rakhshandehroo *et al.*, 2009). However, it increases the susceptibility of transgenic *N. benthamiana* plants to *Plum pox virus* (Ying *et al.*, 2010). Despite some discrepancies in its observed functions, RDR1 is known to act an important factor for virus-derived interfering RNA biogenesis and for antiviral defense (Qi *et al.*, 2009; Qu, 2010).

Interestingly, no convincing role of RDR1 has been reported in the biogenesis of siRNAs during geminivirus infection (e.g. *Cabbage leaf curl virus*) (Aregger *et al.*, 2012). Apart from RDR1, RDR6 also participates in this RNAi-mediated antiviral pathway. For example, *RDR6*-deficient *N. benthamiana* showed an impaired defense response against *Tomato yellow leaf curl China virus* (TYLCCNV). In addition, host specificity was also compromised in the *RDR6* mutant of *A. thaliana*, which assisted in the progression of TYLCCNV infection (Li *et al.*, 2014).

Despite the known functions of RDR6 in RNA silencing, the role of RDR1 has not been investigated extensively, especially against geminiviruses. Our current study elucidates a novel function of RDR1 in providing defense against ToLCGV that leads to host-specific symptom recovery. Enhanced expression of RDR1 is correlated with reduced ToLCGV titer and an enhanced level of methylation of the viral promoter in the recovered leaves. We also demonstrate that selective repression of RDR1 by the pre-coat protein of ToLCNDV is crucial for blocking symptom remission in tobacco.

## Materials and methods

### Plant material

Wild-type plants (*Nicotiana benthamiana* and *N. tabacum* cv. Xanthi) and transgenic *N. benthamiana* overexpressing *NtRDR1* plants were grown on sterile soil and placed in an insect-free growth chamber at 25 ± 2 °C, 60% relative humidity, and 16/8 h (light/dark) photoperiod.

### Viral constructs

The partial tandem repeat constructs of ToLCGV (AY190290 and AY190291) and ToLCNDV (U15015 and U15017) used in this study have been reported previously (Padidam *et al.*, 1995; Chakraborty *et al.*, 2003). To facilitate site-directed mutagenesis, ToLCNDV DNA-A was re-cloned into the pUC18 vector at the *Sac*I site, whilst the available monomeric clone of ToLCGV was used as a template.

### Generation of infectious constructs of the viral mutants and agroinoculation

Point mutations were introduced into the *AC2*, *AC4*, and *AV2* ORFs of ToLCNDV and ToLCGV DNA-A components using a QuikChange Site-Directed Mutagenesis Kit (Stratagene, USA) through overlapping primers (Supplementary Table S1 at *JXB* online). Nonsense codons were introduced in the ORFs to separately generate ∆AC2, ∆AC4, ∆AV2, ∆AC2∆AC4, and ∆AC2∆AV2 mutants of both ToLCNDV and ToLCGV (Table 1). All the mutants were sequenced to confirm the changes in their respective nucleotides. Infectious constructs of all the mutants were developed as described by Chakraborty *et al.* (2003) using appropriate restriction sites followed by agroinoculation of test plants. Briefly, all the constructs were transformed into *Agrobacterium tumefaciens* strain EHA105 and the positive bacterial colonies harboring the infectious clones were subjected to agroinoculation (by the stem inoculation method) (Ranjan *et al.*, 2013; Kushwaha *et al.*, 2015). Inoculated plants were maintained in an insect-proof glasshouse under 16/8-h light/dark period and 25 ± 2 °C until 4 weeks post-inoculation (wpi) in order to assess symptom severity according to the methods described by Chakraborty *et al.* (2008). All the mutations were confirmed by standard sequencing of PCR-amplified *AC1* and *AV2* products from the progeny viral DNA isolated from at least three different plants inoculated with each viral mutant. All the experiments were repeated three times.

**Table 1.** Strategies used to generate various mutants of ToLCNDV and ToLCGV. Mutants were generated by introducing the stop codons in viral ORFs through side-directed mutagenesis

Mutants	Codon and position of amino acid replacement in virus genomes	
	ToLCNDV	**ToLCGV**
∆AC2	Serine, 5th	Serine, 5th
∆AC4	Phenylalanine, 8th	Cysteine, 9th
∆AV2	Tryptophan, 2nd	Tryptophan, 2nd
∆AC2∆AC4	Serine, 5th and Phenylalanine, 8th	Serine, 5th and Cysteine, 9th
∆AC2∆AV2	Serine, 5th and Tryptophan, 2nd	Serine, 5th and Tryptophan, 2nd

### Southern hybridization and quantification of viral DNA

Total DNA was extracted from virus- and mock-inoculated plants (Dellaporta *et al.*, 1983). Approximately 10 µg of isolated DNA was separated on 0.8% agarose gel and transferred to a Hybond-N^+^ membrane (Amersham, UK) (Sambrook and Russel, 2001). Viral DNA was detected by hybridizing blots using α-^32^P-labelled ORF *AC1* of ToLCNDV and ToLCGV. Radiolabeling was done using the random oligonucleotide primed synthesis method (Feinberg and Vogelstein, 1983). Detection of viral bands was performed using a Typhoon phosphor image analyser (Amersham, UK) as described by Kumar *et al.* (2015).

### Northern hybridization

Total RNA from the leaves of virus-infected and mock-inoculated plants was extracted and treated with DNaseI for 1 h. Total RNA (10 µg) was separated on 1.2% formaldehyde agarose gel and transferred to a nylon membrane (Amersham, UK). Viral transcripts were detected by hybridizing blots using a α-^32^P-dCTP labeled probe (ORF AC1) of ToLCNDV. Viral transcripts were detected using a Typhoon phosphor image analyzer (Amersham, UK).

### Isolation and detection of siRNAs

Total RNA isolated from 1 g of systemically infected leaves was subjected to enrichment of low molecular weight RNAs by 5% polyethylene glycol and 0.5M NaCl. Enriched RNAs were further separated in a 15% tris-borate-EDTA-urea acrylamide gel and transferred to a Hybond-N^+^ membrane (Amersham, UK) using a semi-dry electro-blotter (Amersham, UK). For detection of virus-specific siRNA, α-^32^P-dCTP labeled DNA probes of overlapping regions between AC1/AC2 and AC2/AC3 of ToLCNDV (nt 1148–1701) and ToLCGV (nt 1209–1550) were used. The sRNA blots were re-probed with miR-160 (5′-TGGCATACAGGGAGCCAGGCA-3′) to serve as a loading control. Hybridization was carried out at 40 °C overnight and siRNAs were detected using a Typhoon phosphor image analyser (Amersham, UK).

### Replacement of ToLCGV-AV2 with ToLCNDV-AV2 and plant inoculation

Restriction sites (*SpeI* and *AvrII*) were introduced before the start codon and after the stop codon of *AV2*, respectively, in the ToLCGV-DNA-A through site-directed mutagenesis. ToLCNDV-AV2 was cloned in place of ToLCGV-AV2 at these sites. The chimeric molecule (VANDAV2) was confirmed by restriction digestion with *EcoR*V and sequencing. A partial tandem repeat of this molecule was generated in pCAMBIA2301 at the *EcoR*I and *Hind*III sites and mobilized into *A. tumefaciens* strain EHA105 for plant inoculation.

### Agroinfiltration

For infiltration, ToLCGV-AV2, ToLCNDV-AV2, and ToLCGV-AC2 ORFs were cloned into the pGR106 vector (Potato virus X based vector) at the *Cla*I and *Not*I sites, and ToLCNDV-AC2 ORF was cloned at the *Not*I and *Sal*I sites in the pGR106 vector. The primers used to generate the corresponding constructs are listed in Supplementary Table S1. Clones were agrotransformed and agroinfiltration was carried out (leaf infiltration) using standard procedures as described previously (Kushwaha *et al.*, 2017).

### Bisulfite sequencing

Bisulfite sequencing was performed to investigate the relative level of cytosine methylation, following Yadav and Chattopadhyay (2011). Total DNA was isolated from ToLCNDV- and ToLCGV-infected leaves of *N. tabacum* (5th leaf at 3 wpi), from ToLCNDV- and ToLCGV-infected leaves of non-transgenic *N. benthamiana* plants (two uppermost leaves at 3 wpi), and from ToLCNDV- and ToLCGV-infected leaves of *NtRDR1*-overexpressing transgenic *N. benthamiana* plants (two uppermost leaves at 3 wpi) and further subjected to digestion with *Sac*I (20 U µl^–1^) and Proteinase K (20 U µl^–1^). Bisulfite conversion of digested and purified DNA was performed using an EZ-DNA Methylation-Gold kit (Zymo Research, USA) according to the manufacturer’s protocol. Virion strand-specific primers were designed from the intergenic region (IR) of ToLCNDV and ToLCGV (see Supplementary Table S1). Amplification of desired fragments was performed using ‘hot-start’ Taq polymerase (5 U µl^–1^) (Zymo Research, USA) followed by cloning of the PCR-amplified products into the pGEM-T Easy vector (Promega, USA). The cloned products were sequenced (ABI Sequencer-3770, Applied Biosystems, USA) and further analysed for the level of DNA methylation.

### Quantitative real time-PCR (qRT-PCR)

Gene-specific primers were designed for *DCL*s, *Argonaut*s (*AGO*s), *RNA-dependent RNA polymerases* (*RDR*s), *dsRNA binding protein* (*DRBP*), and ToLCGV-*AC2*, using the Primer Express 3.0 software (Applied Biosystem, CA; see Supplementary Table S1). RT-PCR was performed on an Eco Real-time PCR system (Illumina, USA) following Kushwaha *et al.* (2015). The threshold cycle (*C*_t_) value corresponding to the host transcripts was normalized with that of the internal control (*Actin*). ∆∆*C*_t_ values were analysed using the PRIZM software (https://www.graphpad.com/scientific-software/prism).

## Results

### Differential responses of *Nicotiana* spp. against tomato-infecting geminiviruses

**Fig. 1.**
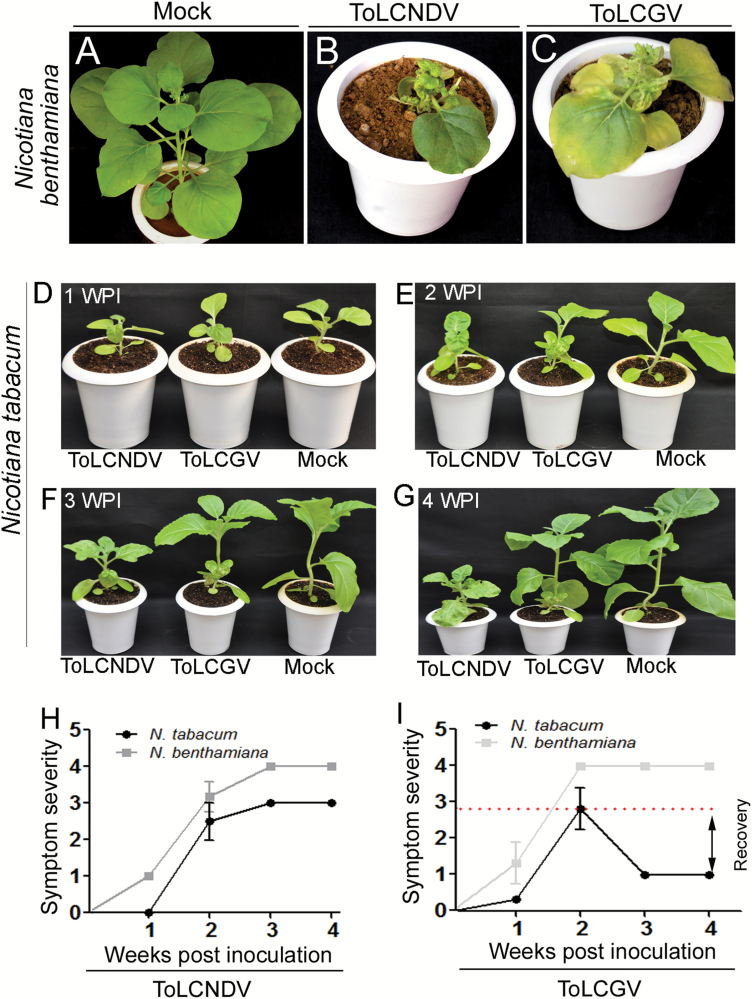
Differential infectivity of ToLCNDV and ToLCGV on *Nicotiana* spp. (A–C) Symptom appearance on *N. benthamiana* inoculated with mock (A), ToLCNDV (B), and ToLCGV (C) at 4 weeks post-inoculation (wpi). (D–G) Comparative phenotypes of *N. tabacum* cv. Xanthi inoculated with either mock, ToLCNDV, or ToLCGV at 1 wpi (D), 2 wpi (E), 3 wpi (F), and 4 wpi (G), indicating progressive disease development. (H, I) Symptom severity of *N. benthamiana* and *N. tabacum* inoculated with ToLCNDV (H) and ToLCGV (I).

Infection with either ToLCNDV or ToLCGV showed systemic symptoms on *N. benthamiana* and *N. tabacum* (cv. Xanthi) plants (Fig. 1A–G). Subsequent to ToLCNDV inoculation, initial symptoms appeared on *N. benthamiana* 3 d earlier than on *N. tabacum* (Fig. 1H, I; Table 2). Both the *Nicotiana* spp. developed severe symptoms following ToLCNDV infection (Fig. 1B, D–G). Nevertheless, disease progression was similar for both hosts, with a steady increase in symptom severity (Fig. 1H). Interestingly, both the species of *Nicotiana* failed to recover from ToLCNDV infection. We also evaluated the infectivity of ToLCGV on both *Nicotiana* spp. (Fig. 1C, D–G), and both hosts were found to be susceptible at the early stages (Table 2). However, ToLCGV-infected *N. tabacum* plants showed characteristic symptom recovery at 2 wpi onwards (Fig. 1D–G, I). Plants of *N. benthamiana* inoculated with ToLCGV developed severe symptoms at 13 d post-inoculation (dpi) and these were persistent throughout the course of study (Fig. 1I). A comparative phenotyping for *N. tabacum* is shown in Supplementary Fig. S1A–H.

**Table 2.** Infectivity of mutants and wild-type of ToLCNDV and ToLCGV in *N. benthamiana and N. tabacum*

Mutants	*N. benthamiana*				*N. tabacum*			
	Symptoms	Days to symptom appearance	Severity	Symptomatic plants/Plants inoculated	Symptoms	Days to symptom appearance	Severity	Symptomatic plants/Plants inoculated
**ToLCNDV**								
∆AC2	LC, ST	9	**+++**	30/33	**–**	**–**	**–**	0/18
∆AC4	LC, ST, YL, SL, CL, CR	6	**++++**	29/33	LC, ST, YL, CL, CR, VB	8	**++++**	15/18
∆AC2∆AC4	LC, ST	13	**++**	25/33	–	–	**–**	14/18
∆AV2	LC, ST, REC	7	**+**	31/33		–	**–**	0/18
∆AC2∆AV2	–	–	**–**	0/33	–	–	**–**	0/18
Wild-type	LC, ST, YL, SL, CR, CL	5	++++	32/33	LC, ST, YL, CL, CR, VB	8	**++++**	15/18
**ToLCGV**								
∆AC2	LC, ST, YL, SL, CL	7	**++++**	16/18	**–**	**–**	**–**	0/18
∆AC4	LC, ST, YL, SL, CL, CR	6	**++++**	16/18	**–**	–	**–**	0/18
∆AC2∆AC4	Very mild LC	22–23	**++**	14/18	–	–	**–**	0/18
∆AV2	LC, ST, REC	6	**+**	15/18		–	**–**	0/18
∆AC2∆AV2	–	–	**–**	0/18	–	–	**–**	0/18
Wild-type	LC, ST, YL, SL, CR, CL	5	++++	18/18	initial LC, ST, YL followed by REC	8	**++++**	29/33

LC, leaf curling; ST, stunting; CR, leaf crumpling; YL, yellowing; CL, chlorosis; SL, small leaf; VB, vein banding; REC, recovery, i.e. decrease in disease symptom severity. Severity of symptoms was scored from mild (+) to severe (++++)

In an attempt to determine whether the recovery phenotype could be reversed, *N. tabacum* plants undergoing recovery (4 wpi) were re-inoculated with either ToLCGV or ToLCNDV. Even after re-inoculation with ToLCGV, newly emerging leaves failed to exhibit symptoms over the duration of the experiment (Supplementary Fig. S1I). Interestingly, recovered plants re-inoculated with ToLCNDV developed severe leaf curl symptoms (Supplementary Fig. S1I). These results suggest that ToLCNDV infection disrupts the recovery phenotype, hence resulting in enhanced disease development in *N. tabacum*.

### Recovered leaves have altered levels of viral DNA, transcripts, and siRNAs

**Fig. 2.**
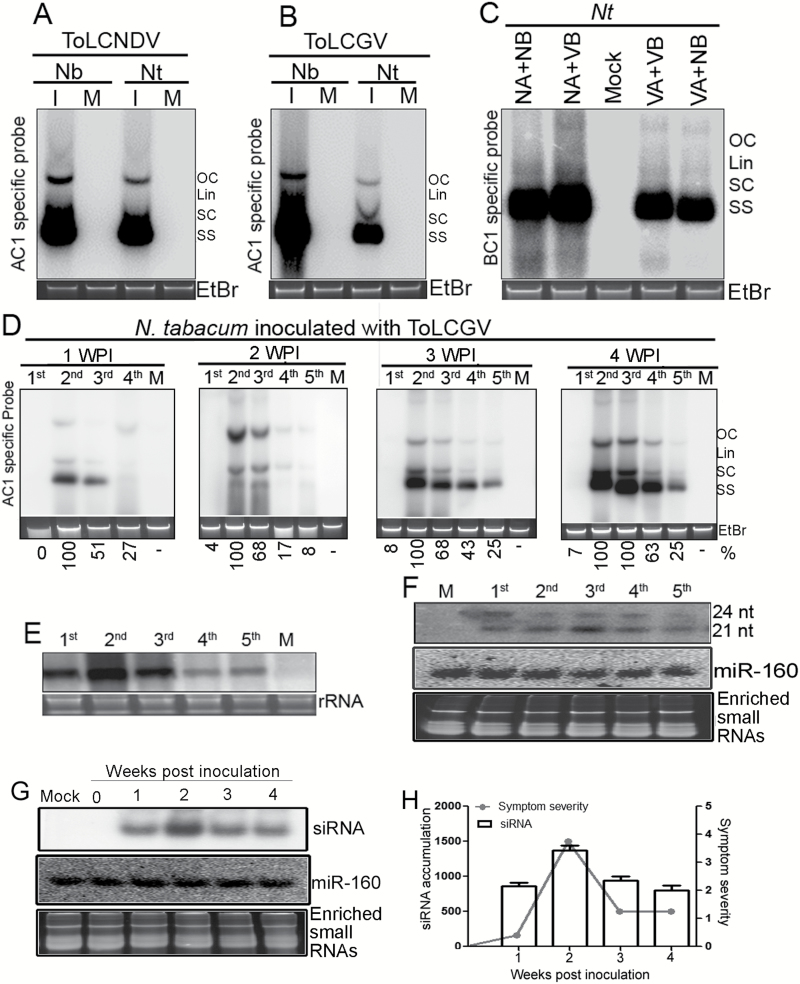
Viral DNA accumulation in *N. benthamiana* (Nb) and *N. tabacum* (Nt) inoculated with ToLCNDV (A) or ToLCGV (B) at 3 weeks post-inoculation (wpi). (C) DNA-B levels in *N. tabacum* inoculated with cognate and heterologous components. (D) Comparative levels of ToLCGV accumulation in 1st, 2nd, 3rd, 4th, and 5th leaves of *N. tabacum* cv. Xanthi at 1, 2, 3 and 4 wpi. Total genomic DNA stained with ethidium bromide (EtBr) at the bottom of each gel serves as the loading control. Four replicative forms of viral DNA are indicated: OC, open circular; Lin, linear; SC, super-coiled; SS, single-stranded. (E) Relative levels of ToLCGV transcript (AC1) in different leaves of *N. tabacum* at 3 wpi. (F) Relative levels of ToLCGV-derived siRNAs in different leaves of tobacco (as indicated in D) at 3 wpi. (G) Detection of ToLCGV-derived siRNA at 1–4 wpi. Ethidium bromide-stained enriched small RNAs and mi-R160 blots are been shown as the loading controls. (H) Relative levels of siRNAs and symptom severity at different stages of disease development.

We further examined the levels of viral DNA in both the species of *Nicotiana* at 3 wpi. The symptomatic leaves inoculated with ToLCNDV contained higher levels of viral DNA (Fig. 2A); however, a considerable decrease in the level of ToLCGV-specific DNA was found in the systemic leaves of *N. tabacum* in comparison with *N. benthamiana* at 3 wpi (Fig. 2B). The role of the DNA-B component of both ToLCNDV and ToLCGV in recovery as well as the levels of viral DNA were also examined. For this, cognate DNA-A of either ToLCNDV or ToLCGV was trans-complemented with heterologous DNA-B to infect *N. tabacum* plants. It was observed that genomic re-assortments between ToLCNDV and ToLCGV did not affect the relative level of viral DNA in comparison with homologous combinations (Fig. 2C). These results suggest that DNA-B has no role in the process of recovery against ToLCGV infection.

The viral DNA levels in the ToLCGV-infected leaves of each of the test plants were evaluated to study the time-specific progression of recovery in *N. tabacum* (Fig. 2D; Supplementary Fig. S2A). Higher levels of viral DNA were observed in the 2nd and 3rd systemically infected leaves at 2 to 4 wpi (Fig. 2D). Interestingly, the 4th and 5th leaves contained relatively less amounts of ToLCGV-specific DNA at all the time points (Fig. 2D). We determined that the systemically infected part of *N. tabacum* corresponding to the 3rd, 4th, and 5th leaves had relatively enriched accumulation of ToLCNDV at 3 wpi (Supplementary Fig. S2B). Moreover, the *AC1*-specific transcript of ToLCGV was also found to be up-regulated in the first three systemically infected leaves, in comparison to the 4th and 5th leaves (Fig. 2E). The accumulation of ToLCGV-specific siRNAs was investigated in systemically-infected leaves of *N. tabacum*, and it was observed that all the leaves contained ToLCGV-specific siRNAs at 3 wpi (Fig. 2F). A significant correlation between viral-specific siRNA accumulation and symptom severity was observed in ToLCGV-inoculated *N. tabacum* plants (Fig. 2G, H). Moreover, ToLCNDV-infected leaves of *N. tabacum* showed lower levels of virus-specific siRNAs (Supplementary Fig. S2C).

### AV2 is the major pathogenicity factor of both tomato-infecting geminiviruses

**Fig. 3.**
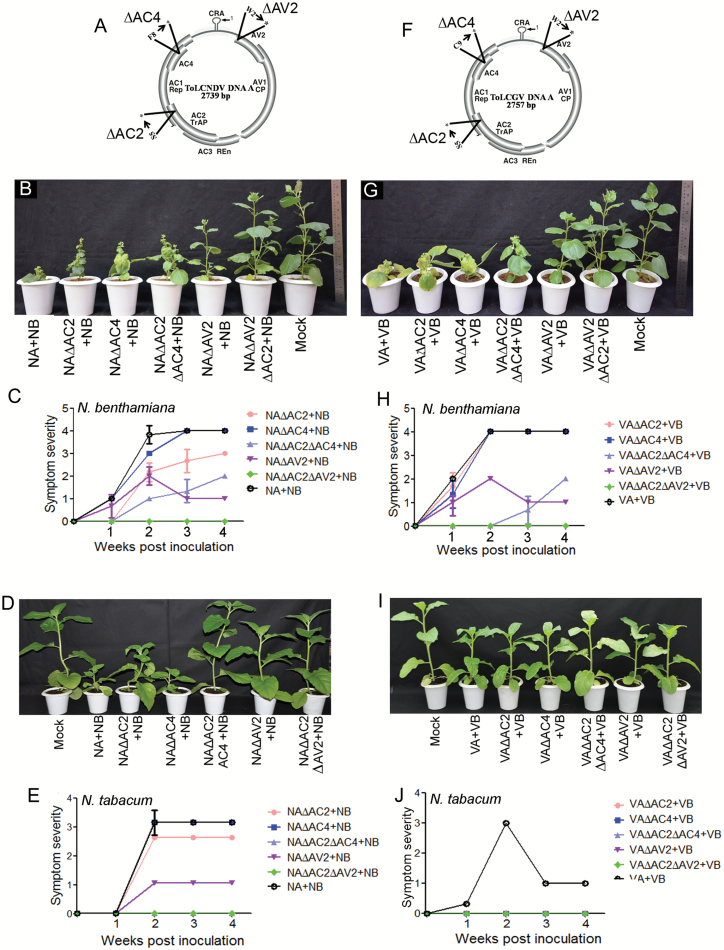
Infectivity of viral mutants on *Nicotiana* spp. (A) Schematic representation of various mutations introduced in ToLCNDV infectious clones. In ∆AC2 the 5th amino acid serine (S5) was replaced with a stop codon (*); in ∆AC4 the 8th amino acid phenylalanine (F8) was replace with a stop codon (*). The pre-coat protein AV2 was mutated by introduction of a stop codon in place of tryptophan at the second position (W2) to generate ∆AV2. The double-mutants ∆AC2∆AC4 and ∆AC2∆AV2 were also generated through the same procedure. (B) Phenotypes and (C) the symptom severity of *N. benthamiana* inoculated with the mutants and the wild-type of ToLCNDV at 3 weeks post-inoculation (wpi). (D) Phenotype of *N. tabacum* inoculated with the mutants and the wild-type construct. (E) Symptom severity of *N. tabacum* plants inoculated with the mutants and the wild-type construct. (F) Schematic representation of various mutations in ToLCGV. A similar strategy was applied to generate mutants as described in (A). (G) Phenotypes and (H) the symptom severity of *N. benthamiana* inoculated with the mutants and the wild-type at 3 wpi. (I) Phenotypes of *N. tabacum* inoculated with mutants and the wild-type construct. (J) Symptom severity of *N. tabacum* plants inoculated with mutants and the wild-type construct.

The explicit roles of individual viral ORFs in pathogenesis were determined by introducing in-frame stop codons either in a single ORF (AC2, AC4, and AV2) or in double ORFs (AC2AC4 and AC2AV2) of ToLCNDV-DNA-A (Fig. 3A). Both *Nicotiana* spp. were co-inoculated with either wild-type or mutant DNA-A along with the cognate DNA-B component (Table 2). A nonsense mutation in AC2 ORF (∆AC2) resulted in delayed and milder development of symptoms on *N. benthamiana* in comparison with the wild-type (Fig. 3B, C). On the other hand, *N. tabacum* plants inoculated with ∆AC2 showed milder symptoms as compared to the wild-type virus. ∆AC4-inoculated plants of both *Nicotiana* spp. exhibited severe symptoms (Fig. 3B, D). ∆AV2-inoculated *N. benthamiana* showed a characteristic symptom-remission phenotype from 2 wpi onwards (Fig. 3C). The effect of the ∆AC2∆AC4 double-mutation on *N. benthamiana* was to cause delayed and attenuated symptoms, while *N. tabacum* failed to develop any visible symptoms (Fig. 3B–E). Similarly, plants inoculated with ∆AC2∆AV2 did not show any symptoms of disease (Fig. 3B–E).

A similar strategy was used to generate mutants of ToLCGV DNA-A (Fig. 3F). It was observed that *N. benthamiana* plants inoculated with either ∆AC2 or ∆AC4 exhibited severe symptoms of virus infection at later stages, but ∆AV2 resulted in declining symptom severity from 2 wpi onwards (Fig. 3G, H; Table 2). In contrast, *N. tabacum* plants inoculated with either of these mutant constructs remained asymptomatic at 4 wpi (Fig. 3I, J). Plants of *N. benthamiana* were weakly susceptible to the ∆AC2∆AC4 mutant and exhibited delayed and attenuated symptoms (Fig. 3G, H), whilst *N. tabacum* plants did not exhibit any noticeable symptoms after agroinoculation with ∆AC2∆AC4 (Fig. 3I, J). The other double-mutant, ∆AC2∆AV2, failed to infect either of the host plants (Fig. 3G–J).

### Mutations in ORFs lead to the differential accumulation of virus-specific DNA and siRNAs

**Fig. 4.**
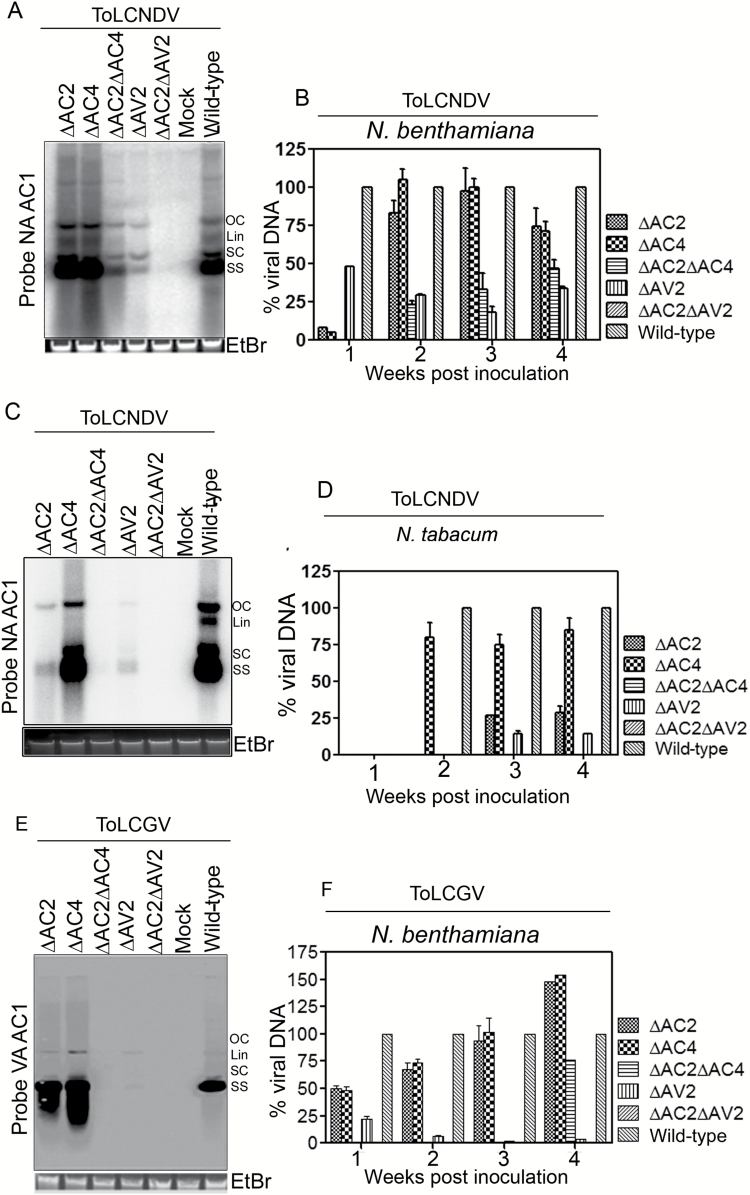
Relative levels of viral DNA accumulation in *Nicotiana* spp. (A) Viral DNA abundance in *N. benthamiana* plants inoculated with mutants and wild-type ToLCNDV at 3 weeks post-inoculation (wpi). (B) Viral DNA accumulation (%) in *N. benthamiana* plants inoculated with mutants and the wild-type ToLCNDV at 1–4 wpi. (C) Viral DNA abundance in *N. tabacum* plants inoculated with various mutants and wild-type ToLCNDV at 3 wpi. (D) Viral DNA accumulation (%) in *N. tabacum* inoculated with mutants and wild-type ToLCNDV at 1–4 wpi. (E) Viral DNA abundance in *N. benthamiana* inoculated with mutants and wild-type ToLCGV at 3 wpi. (F) Viral DNA accumulation (%) in *N. benthamiana* inoculated with mutants and wild-type ToLCGV. The probes used for viral detection are given beside each blot. Four replicative forms of viral DNA are indicated: OC, open circular; Lin, linear; SC, super-coiled; SS, single-stranded. EtBr, ethidium bromide.

The effect of mutations on accumulation of either ToLCNDV or ToLCGV was studied by comparing the level of viral DNA in wild-type samples of the *Nicotiana* spp. at 1–4 wpi (Fig. 4A–F). The accumulation of wild-type viral DNA was considered as maximum (100%). ∆AC2-inoculated *N. benthamiana* had the highest accumulation of ToLCNDV DNA (~102%) at 3 wpi (Fig. 4A, B), whilst ∆AV2 infection in *N. benthamiana* resulted in the highest accumulation (~48%) of ToLCNDV DNA at 1 wpi (Fig. 4A, B). Plants infected with the ∆AC2∆AC4 double-mutant showed relatively lower accumulation of viral DNA at all stages of ToLCNDV infection in comparison with the wild-type virus in *N. benthamiana* (Fig. 4A, B). Contrasting levels of ToLCNDV-DNA were observed in *N. tabacum* plants inoculated with the different virus mutants, with the ∆AC4, ∆AC2, and ∆AV2 mutants accumulating detectable levels at 2–4 wpi (Fig. 4C, D). Plants of *N. benthamiana* inoculated with either the ∆AC2 or ∆AC4 mutant of ToLCGV accumulated to a similar level as the wild-type at 3 wpi (Fig. 4E, F).

We also evaluated the effect of inoculation with the mutants on DNA-B-specific DNA accumulation in *N. benthamiana* (Supplementary Fig. S3A, B). It was observed that the ∆AC2, ∆AC4, and ∆AC2∆AC4 mutants of ToLCNDV-inoculated plants had substantial reduction in accumulation of the DNA-B component compared with the wild-type (Supplementary Fig. S3A). Interestingly, DNA-B-specific accumulation was not detected in plants inoculated with either ∆AV2 alone or with the ∆AC2∆AV2 double-mutant. Plants of *N. benthamiana* probed with ToLCGV-*BC1* showed a similar pattern of DNA-B abundance in ∆AC2- and ∆AC4-inoculated plants, whereas no DNA-B specific molecules were detected in plants inoculated with ∆AC2∆AC4 (Supplementary Fig. S3B). Inoculation with ∆AV2 and ∆AC2∆AV2 did not yield detectable levels of ToLCGV-DNA-B molecules in *N. benthamiana* (Supplementary Fig. S3B). Examination of DNA-B accumulation in *N. tabacum* showed that plants were deficient in DNA-B-specific molecules, except for wild-type ToLCGV-inoculated plants (Supplementary Fig. S3C, D). Comparative analysis of accumulation in the two *Nicotiana* spp. showed that both the DNA-A and DNA-B components had a similar pattern of accumulation in plants inoculated with mutants of either ToLCNDV or ToLCGV.

We quantified the relative level of ToLCNDV-specific siRNAs from systemic leaves of host plants inoculated with both the wild-type and mutants of ToLCNDV at 3 wpi. For reference, accumulation in the wild-type was considered as 100%. Considerable abundance of ToLCNDV-specific siRNAs was observed in ∆AC2-, ∆AC4-, and ∆AC2∆AC4-inoculated *N. benthamiana* plants from 2 wpi onwards, and accumulation was highly abundant in the ∆AV2-inoculated plants at 3 wpi (Supplementary Fig. S4A, D). Inoculation with ∆AC4 resulted in enriched siRNA accumulation (79%) in *N. benthamiana* at 4 wpi (Supplementary Fig. S4D). In contrast, *N. tabacum* inoculated with the ToLCNDV mutants failed to accumulate detectable level of siRNAs (Supplementary Fig. S4B, E). The ∆AC4 mutant had relatively higher accumulation (>70%) of siRNAs in comparison with other mutants at 2–4 wpi (Supplementary Fig. S4B, E). Examination of the relative levels of ToLCGV-specific siRNAs in *N. benthamiana* inoculated with the mutants revealed that ∆AC2- and ∆AC4-inoculated plants showed similar levels of siRNA production through all the experimental time points. Maximum levels (>160%) of siRNAs were observed at 3 wpi (Supplementary Fig. S4C, F).

### Involvement of AV2 of tomato-infecting geminiviruses in host recovery

**Fig. 5.**
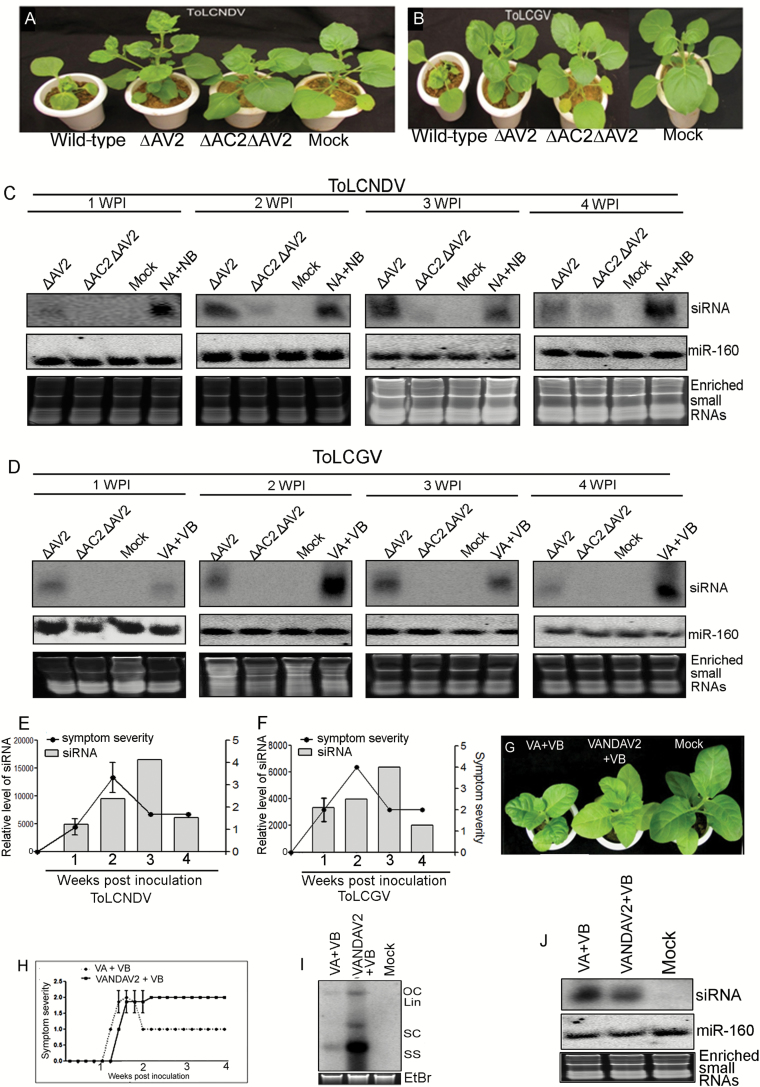
Relationship between symptom severity, recovery, and siRNA accumulation associated with AV2 mutants. Phenotype of *N. benthamiana* plants inoculated with wild-type, ΔAV2, and ΔAC2ΔAV2 mutants of (A) ToLCNDV and (B) ToLCGV at 3 weeks post-inoculation (wpi). Relative levels of viral siRNA in *N. benthamiana* plants inoculated with ΔAV2, ΔAC2ΔAV2, and wild-type of (C) ToLCNDV and (D) ToLCGV at 1–4 wpi. Correlation of symptom severity with siRNA accumulation in ΔAV2 for (E) ToLCNDV and (F) ToLCGV at different infection stages. (G) Phenotypes of *N. tabacum* plants inoculated with either wild-type or chimeric DNA molecules (replacing AV2 of ToLCGV with ToLCNDV-AV2) of ToLCGV together with the DNA B (VB) component. (H) Symptom severity on *N. tabacum* plants inoculated with either wild-type (VA+VB) or chimeric ToLCGV (VANDVAV2+VB). Relative levels of (I) viral DNA and (J) viral-specific siRNA accumulation on *N. tabacum* plants inoculated with wild-type and chimeric ToLCGV at 3 wpi.

In order to identify correlations between symptom severity and RNAi, we compared the phenotype and siRNA production of *N. benthamiana* following inoculation with ToLCNDV- and ToLCGV-specific mutants (∆AV2 and ∆AC2∆AV2) (Fig. 5A–F). AV2 was identified as a major determinant of pathogenicity for both the begomoviruses. In ΔAV2-inoculated *N. benthamiana* plants, symptom remission started from approximately 2 wpi onwards (Fig. 5A, B, E, F). Accumulation of both ToLCNDV- and ToLCGV-specific siRNAs were maximum (>80%) at 3 wpi, and this decreased to 50% during the later stages of infection (Fig. 5C, D). Increases in the levels of siRNAs were observed that corresponded with the declines in the symptom severity (Fig. 5E, F). Hence, a strong negative correlation between symptom severity and siRNA accumulation in *N. benthamiana* was observed.

### AV2 of ToLCNDV can selectively block symptom recovery in *N. tabacum*

Inoculation of *N. tabacum* with a chimeric molecule (VANDAV2) together with ToLCGV-DNA-B was carried out to assess possible correlations between AV2-specific inhibitions of symptom recovery (Fig. 5G–J). The result was a delayed onset of symptoms in comparison with plants inoculated with the wild-type virus (ToLCGV) (Fig. 5G, H), with the maximum severity being reached at ~2 wpi and remaining constant thereafter (Fig. 5H). An increase in the level of ToLCGV-DNA (by ~5-fold) was observed in symptomatic leaves of *N. tabacum* inoculated with the chimeric virus (Fig. 5I). A decreased level of viral siRNA (~30%) was measured in systemic leaves of *N. tabacum*, in contrast to the corresponding recovered leaves inoculated with the wild-type virus (Fig. 5J). These results indicated that ToLCNDV-AV2 could selectively block recovery in tobacco.

### Enhanced levels of RDR1 are linked to recovery from ToLCGV infection in *N. tabacum*

**Fig 6.**
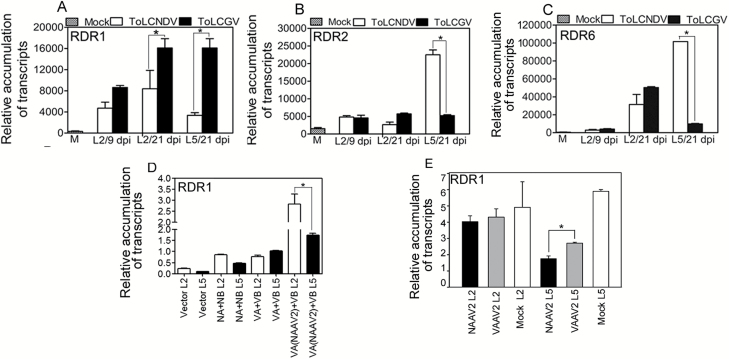
Relative quantification of transcripts of RDRs in *N. tabacum*. qRT-PCR analysis of (A) *RDR1*, (B) *RDR2*, and (C) *RDR6* in *N. tabacum* inoculated with either ToLCNDV or ToLCGV. Leaf samples are denoted as L2/9dpi (2nd leaf at 9 dpi), L2/21dpi (2nd leaf at 21 dpi) and L5/21dpi (5th leaf at 21 dpi). (D) Levels of *NtRDR1* transcripts in L2 and L5 of *N. tabacum* plants inoculated with ToLCNDV (NA+NB), ToLCGV (VA+VB), and ToLCGV containing chimeric molecules together with the vector control at 3 weeks post-inoculation (wpi). (E) Relative accumulation of RDR1 transcripts in *N. tabacum* infiltrated with either PVX (mock), PVX-ToLCNDV-AV2 (NAAV2), or PVX-ToLCGV-AV2 (VAAV2) at 3 wpi. Data are means (±SD) of three biological replicates. Relative mRNA levels were determined by qRT-PCR using a standard curve approach, and the value of each biological repeat is the mean of three technical repeats. All values are normalized with respect to the internal control, *Actin*. *P<0.001 (Student’s *t*-test).

Relative transcript accumulations of *AGO*s, *DCL*s, *DRBP*, *RDRP*s, and *SGS3* were investigated to identify the possible role of major components of the host RNAi machinery in virus-specific symptom recovery. The analysis focused on the 2nd leaf at 9 days post inoculation (dpi) prior to recovery (L2/9dpi), the symptomatic 2nd leaf at 21 dpi (L2/21dpi), and the recovered/non-recovered (corresponding to ToLCGV/ToLCNDV) 5th leaves at 21 dpi (L5/21dpi). The relative accumulation of these host transcripts was compared in *N. tabacum* inoculated with either ToLCGV or ToLCNDV (Fig. 6A–C, Supplementary Fig. S5).

At the later stage (L5/21dpi) of either ToLCNDV or ToLCGV infection, significant (*P*<0.001) differences in the levels of *AGO1* and *AGO2* were observed. ToLCNDV-infected leaves showed relatively higher abundance of these transcripts in comparison to ToLCGV inoculation (Supplementary Fig. 5SA, B). In addition, *AGO4* transcripts were significantly (*P*<0.001) up-regulated in each test sample during the course of ToLCNDV infection, in contrast to ToLCGV-inoculated leaves of *N. tabacum* (Supplementary Fig. S5C). Similarly, *AGO7* transcripts showed significantly (*P*<0.001) higher accumulation at L5/21dpi with ToLCNDV inoculation in comparison to the ToLCGV treatment (Supplementary Fig. S5D). Overall, significantly (*P*<0.001) reduced levels of *AGO*-specific transcripts were observed in the recovered leaves (L5/21dpi) of *N. tabacum* inoculated with ToLCGV (Supplementary Fig. S5A–D).

A significant (*P*<0.001) difference in the accumulation of *SGS3* transcripts was observed in L2/9dpi samples of *N. tabacum* inoculated with ToLCGV and ToLCNDV (Supplementary Fig. S5E). Expression of *DRBP* transcripts was significantly (*P*<0.001) enhanced in the non-recovered leaves (L5/21dpi) infected with ToLCNDV (Supplementary Fig. S5F). In addition, ToLCGV-infected recovered tissue (L5/21dpi) showed decreased levels of *DRBP*-specific transcripts in *N. tabacum* (Supplementary Fig. S5F). The level of *DCL1* transcripts was found to be significantly (*P*<0.001) higher in the L2/21dpi leaves infected with ToLCGV in comparison with ToLCNDV inoculation; however, the pattern was reversed and levels were significantly (*P*<0.001) reduced in ToLCGV-inoculated plants at the later stage (L5/21dpi) (Supplementary Fig. S5G). *DCL2* and *DCL3* showed significant (*P*<0.001) up-regulation in the leaves corresponding to L2/9 dpi and L2/21dpi upon ToLCNDV inoculation, in contrast to ToLCGV inoculation (Supplementary Fig. S5H, I). Differences in the levels of *DCL4*-specific transcripts were not significant in the leaves of *N. tabacum* inoculated with either ToLCNDV or ToLCGV (Supplementary Fig. S5I).

Accumulation of *RDR1* transcripts was significantly (*P*<0.001) higher in L2 and L5 at 21 dpi in plants inoculated with ToLCGV in comparison with ToLCNDV (Fig. 6A). Relative expression of *RDR2* transcripts was significantly (*P*<0.001) reduced in L5 at 21 dpi in plants inoculated with ToLCGV compared with ToLCNDV inoculation (Fig. 6B). Significant up-regulation (*P*<0.001) in the levels of *RDR6* transcripts was detected in the systemic (5th) leaves of *N. tabacum* inoculated with ToLCNDV in comparison with recovered leaves of ToLCGV-inoculated plants at 21 dpi (Fig. 6C). Significant up-regulation of *RDR1* suggests the possible role of this gene in modulating symptom recovery in *N. tabacum* systemic leaves inoculated with ToLCGV.

### ToLCNDV-AV2 interferes with RDR1-mediated antiviral pathways in *N. tabacum*

We observed significant up-regulation of *RDR1* levels in the recovered tissues of *N. tabacum* (Fig 6A) and recovery was diminished upon ToLCNDV infection in those leaf tissues that had relatively low levels of *RDR1* transcripts. Hence, we aimed to evaluate the involvement of ToLCNDV factors in the inhibition of RDR1-mediated recovery. For this, the expression of *NtRDR1* was assessed in *N. tabacum* leaves (2nd and 5th) infiltrated with ToLCNDV (NA+NB), ToLCGV (VA+VB) and with ToLCGV containing ToLCNDV-AV2 (VA[NAAV2]+VB), together with the empty vector control (Fig. 6D).

Samples of the 2nd leaf of plants inoculated with VA[NAAV2]+VB showed significantly (*P*<0.001) higher accumulation of *NtRDR1*, in contrast to 5th leaf at 21 dpi (Fig. 6D). Differences in the levels of *NtRDR1* were significant in the 5th leaf of NA+NB-inoculated plants compared with the corresponding 2nd leaf (Fig. 6D). Infiltration with ToLCGV (VA+VB) resulted in insignificant changes in the expression of *NtRDR1* transcripts in the 5th leaf compared with the 2nd leaf. Hence, the presence of NAAV2 decreased the relative level of *NtRDR1* in the upper systemic leaf.

We infiltrated *N. tabacum* to investigate the effect of PVX (mock), PVX-ToLCNDV-AV2 (NAAV2), and PVX-ToLCGV-AV2 (VAAV2) on the relative expression of *RDR1* at 21 dpi. The expression of *NtRDR1* was reduced significantly (*P*<0.001) in the 5th leaf infiltrated with NAAV2 in comparison with VAAV2. However, no significant differences were observed in 2nd leaves infiltrated with either mock, NAAV2, or VAAV2 (Fig. 6E). These results further suggest that NAAV2 selectively blocks the symptom recovery by down-regulating *RDR1* expression.

### ToLCNDV-∆AV2 failed to infect transgenic *N. benthamiana* plants overexpressing *NtRDR1*

**Fig 7.**
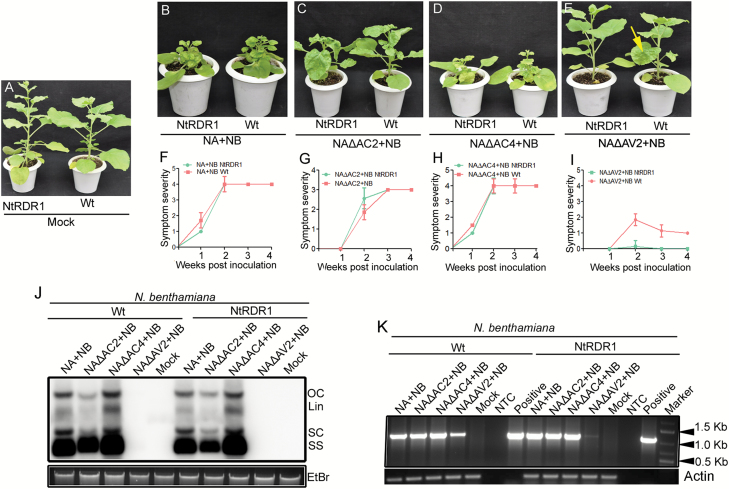
Comparative symptom appearance, viral DNA accumulation, and kinetics of symptom development on wild-type and the *NtRDR1*-overexpression line of *N. benthamiana*. Plants were inoculated with either wild-type or mutants of ToLCNDV. (A–E) Symptoms on wild-type and *NtRDR1*-overexpression *N. benthamiana* plants inoculated with (A) mock, (B) wild-type ToLCNDV (NA+NB), (C) NA∆AC2+NB, (D) NA∆AC4+NB, and (E) NA∆AV2+NB. (F–I) The progress of symptom development on wild-type and the *NtRDR1*-overexpression line of *N. benthamiana* plants inoculated with either wild-type ToLCNDV or mutants, as indicated. The arrow in (E) indicates symptomatic lower leaves of a representative wild-type *N. benthamiana* plant inoculated with NA∆AV2. (J) Southern blot showing comparative viral DNA abundance both wild-type and transgenic in line of *N. benthamiana* plants infected with ToLCNDV and its mutants at 3 weeks post-inoculation (wpi). The bottom panel shows ethidium bromide-stained total genomic DNA as the loading control. (K) Detection of viral DNA accumulation through PCR in wild-type and the *NtRDR1*-overexpression line of *N. benthamiana* inoculated with ToLCNDV and mutants at 3 wpi. Viral DNA was detected in plants infected with NA∆AV2, which was below the detection level of Southern blotting. The non-template control (NTC) was selected as the negative control for the PCR, and the infectious clone of ToLCNDV was used as the positive control. *Actin* served as the internal control.

To further investigate the role of NtRDR1 in ToLCNDV pathogenesis, we inoculated wild-type and *NtRDR1*-overexpressing *N. benthamiana* (NbNtRDR1) plants with ToLCNDV and its mutants (Fig. 7A–E). Both the wild-type and NbNtRDR1 plants showed similar patterns of symptoms, which reached maximum severity (symptom score 4) at 2 wpi (Fig. 7B, F). Infection of NA∆AC2 on wild-type and transgenic NbNtRDR1 plants also resulted in comparable disease phenotypes (Fig. 7C, G), and both showed severe symptoms and similar patterns of disease progression (Fig. 7D, H). Interestingly, when plants were inoculated with NA∆AV2, significant differences in symptom severity were observed, with the NbNtRDR1 plants remaining symptomless but the wild-type *N. benthamiana* showing mild symptoms initially, followed by symptom recovery (Fig. 7E, I), similar to the observations shown in Fig. 3C. Concurrent with the symptom severity, agroinoculation of either the wild-type virus or mutants (NA∆AC2 and NA∆AC4) produced comparable levels of viral DNA in the wild-type and NbNtRDR1 plants (Fig. 7J). Since viral DNA accumulation was below the detection level of Southern blotting in both the wild-type and transgenic plants agroinoculated with NA∆AV2, semi-quantitative PCR was performed with ToLCNDV-*AC1*-specific primers (Fig. 7K). The results indicated that the wild-type plants contained higher levels of viral DNA when compared to NbNtRDR1 plants inoculated with the NA∆AV2 mutant (Fig. 7K).

### Overexpression of *NtRDR1* in *N. benthamiana* leads to symptom remission induced by ToLCGV

**Fig 8.**
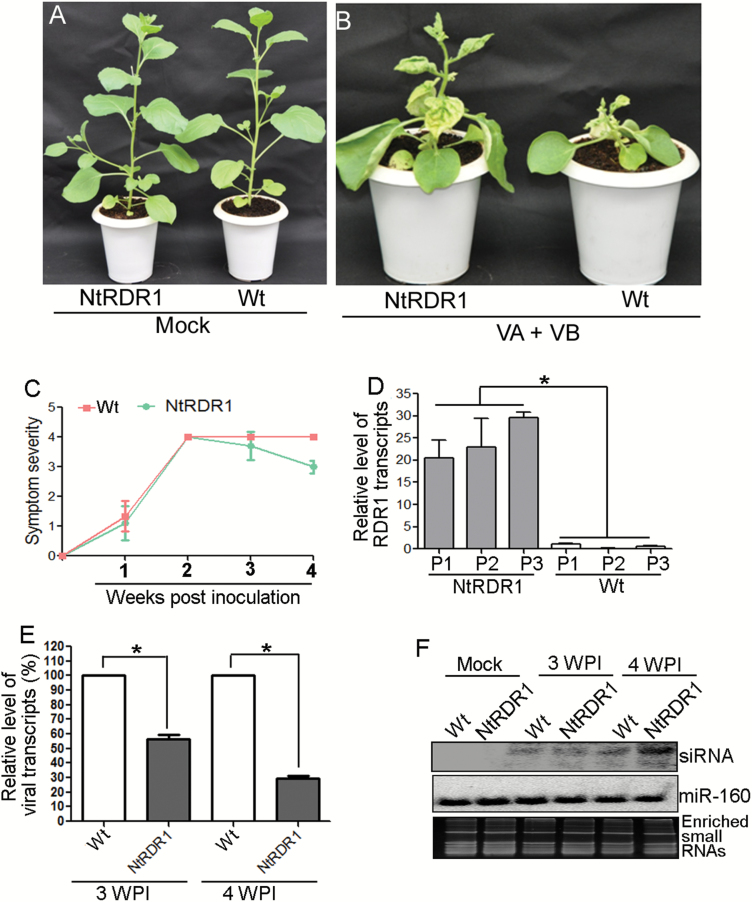
Relative symptoms, progress of symptom development, and viral DNA accumulation in wild-type and *NtRDR1*-overexpression plants of *N. benthamiana* inoculated with (A) mock and (B) VA+VB. (C) Kinetics of symptom development on wild-type and *NtRDR1*-overexpression plants of *N. benthamiana* inoculated with VA+VB. (D) Relative level of *RDR1* transcripts in *NtRDR1*-overexpression and wild-type plants of *N. benthamiana*. (E) Relative accumulation of ToLCGV transcripts (*AC2*) in *NtRDR1*-overexpressing and wild-type plants of *N. benthamiana* at 3 and 4 weeks post-inoculation (wpi). (F) Comparative levels of virus-specific siRNAs at 3 and 4 wpi. miR-160 and enriched small RNA serve as loading controls. *P<0.001 (Student’s *t*-test).

To test whether NbNtRDR1 plants mimic the recovery phenotype (as observed on *N. tabacum* when inoculated with ToLCGV), both wild-type *N. benthamiana* and NbNtRDR1 over-expression plants were agroinoculated with ToLCGV. Non-transgenic plants inoculated with ToLCGV (VA+VB) showed initial leaf curling symptoms from 1 wpi onwards, reached a maximum severity level at 2 wpi, and maintained severity through to 4 wpi (Fig. 8B, C). Notably, NbNtRDR1 transgenic plants inoculated with VA+VB also exhibited a similar disease-development pattern until 3 wpi, after which persistent symptom remission occurred (Fig. 8B, C). NbNtRDR1 transgenic plants contained significantly higher levels of *NtRDR1* transcripts as compared to wild-type *N. benthamiana* plants (Fig. 8D). Further accumulation of viral transcripts was checked at two different time points, corresponding to the onset of recovery in transgenic plants (3 wpi) and to significantly recovered plants (at 4 wpi). The level of ToLCGV-*AC2* transcripts in wild-type *N benthamiana* plants was considered as 100%. The results indicated that NbNtRDR1 transgenic plants contained lower levels of viral transcripts as compared to the wild-type at both the time points (~50% at 3 wpi and ~70% at 4 wpi) (Fig. 8E). Viral-specific siRNAs were found to be more abundant in NbNtRDR1 transgenic plants that had undergone recovery (4 wpi) (Fig. 8F).

### NtRDR1 enhances DNA methylation of the ToLCGV promoter

**Fig. 9.**
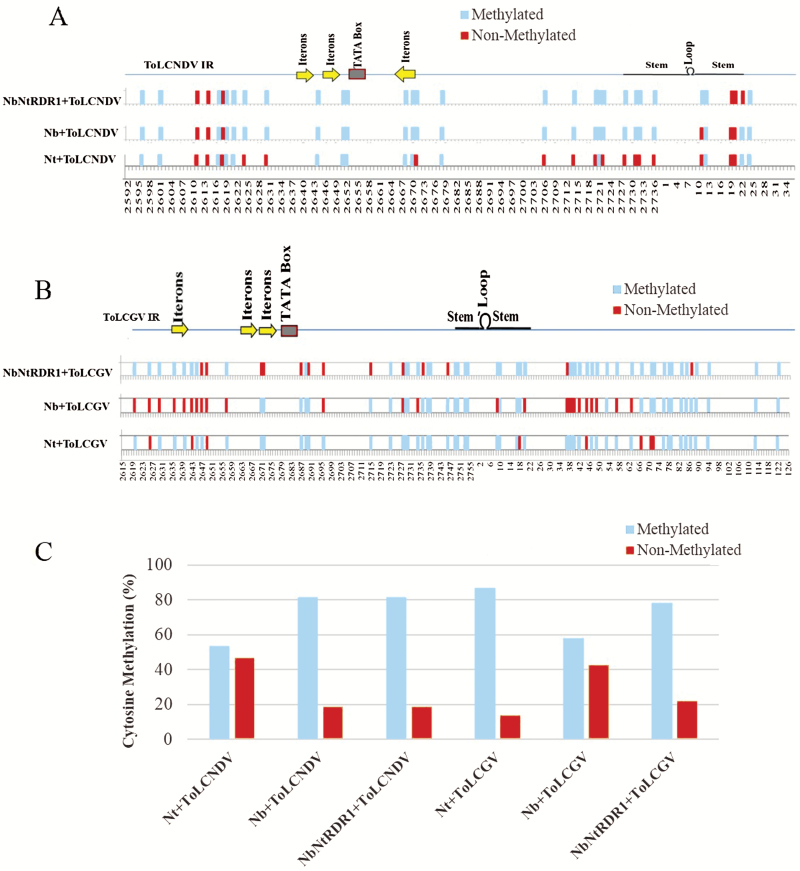
Distribution of viral DNA-methylation in *Nicotiana* spp. infected with tomato-infecting begomoviruses. Schematic representations of cytosine methylation patterns throughout the DNA-A specific intergenic regions (IR) of (A) ToLCNDV and (B) ToLCGV. The positions of methylated and non-methylated cytosine along the IR are indicated. Regulatory elements of the IRs such as iterons, the TATA-box, and stem-loop regions are also illustrated. (C) Relative (%) levels of methylated and non-methylated cytosine in the IRs of ToLCNDV and ToLCGV. Nt, *Nicotiana tabacum*; Nb, *N. benthamiana*; NbNtRDR1, *NtRDR1*-overexpression line of *N. benthamiana*.

We examined the status of DNA methylation of the viral genome in ToLCNDV- and ToLCGV-infected *N. tabacum* at 3 wpi. Bisulfite sequencing-based analysis of five randomly selected clones from each set of experiments showed considerable differences in the level of cytosine methylation between the intergenic regions (IRs) of ToLCNDV and ToLCGV (Fig. 9A–C). ToLCNDV-IR contained 32 cytosines, out of which 17 (53.12%) were found to be methylated, while the remainder (46.88%) were not methylated (Fig. 9A, C, Supplementary Fig. S6).

Fragments corresponding to ToLCGV-IR were analysed by aligning the randomly selected bisulfite-converted DNA from the ToLCGV-infected systemic tissues of *N. tabacum* at 3 wpi. The fragments corresponding to ToLCGV-IR were more heavily methylated than ToLCNDV-IR (Fig. 9A–C). Targeted regions of ToLCGV-IR contained 59 cytosines, out of which 51 (~86%) were found to be methylated (Fig. 9B, C, Supplementary Fig. S7). Regulatory regions such as iterons and the stem-loop present within ToLCGV-IR were hyper-methylated in the ToLCGV-infected leaves as compared to ToLCNDV in *N. tabacum* (Fig. 9B).

To assess the effects of NtRDR1 in the DNA methylation event, we performed bisulfite sequencing of both ToLCNDV-IR and ToLCGV-IR in wild-type and a NtRDR1-overexpression line of *N. benthamiana*. Among the cytosines present in the ToLCNDV-IR, around 80% (26) were found to be methylated in both the wild-type and the NtRDR1-overexpression line (Fig. 9A, C, Supplementary Figs S8, S9); however, in the case of ToLCGV inoculation, the level of cytosine methylation was increased in the NtRDR1-overexpression line. In comparison to wild-type *N. benthamiana*, the NtRDR1-overexpression line showed 20% enhanced methylation (Fig. 9B, C; Supplementary Figs S10, S11). Out of 59 cytosines, 34 were methylated in the wild-type *N. benthamiana* tissues, whilst overexpression of NtRDR1 helped to increase the number of methylated cytosines up to 46.

## Discussion

In the present study, the pathogenesis of ToLCNDV and ToLCGV was investigated on *N. benthamiana* and *N. tabacum*. A steady increase in the symptom severity was recorded in *N. benthamiana* infected with either of these begomoviruses. Symptom recovery of *N. tabacum* following ToLCGV infection was observed, in contrast to plants infected with ToLCNDV. We further aimed to identify the mechanisms behind the species-specific responses of the two *Nicotiana* spp. against ToLCGV. Geminivirus-induced symptom recovery has been reported in diverse plant species (Chellapan *et al.*, 2004; Hagen *et al.*, 2008; Rodríguez-Negrete *et al.*, 2009). Hence, we initially compared the phenotype and viral DNA in both *Nicotiana* spp. and found that the accumulation of viral DNA was in accordance with the symptom severity. The level of viral DNA differed in systemic leaves of *N. tabacum* as disease progressed. Comparative evaluation of viral DNA levels in the leaves adjacent to ToLCGV inoculation suggested that upper systemic leaves had the lowest accumulation of viral DNA. Hence, these leaves could be targeted for antiviral RNA silencing activity.

Viruses are equipped with components that restrict the host’s RNA-silencing activity and this helps them to multiply and spread within the host. In this context, our study clearly establishes the role of ToLCNDV-AV2 as pathogenicity determinant, and it is crucial for suppression of RNAi in the two *Nicotiana* species. As well as ToLCNDV-AV2, ToLCNDV-AC2 and ToLCGV-AV2 were also found to contribute significantly towards disease development. This presumably occurs through suppression of the host anti-viral silencing mechanism. Unlike ToLCNDV-AV2, ToLCGV-AV2 was been found to play an insignificant role in pathogenesis in *N. tabacum*. Further observations revealed that using a chimeric molecule (VANDAV2) to replace ToLCGV-AV2 with ToLCNDV-AV2 also impaired the symptom remission process in *N. tabacum*.

Inconsistency in terms of correlation between leaf-specific accumulation of siRNAs and symptom remission was observed in the recovered leaves (4th and 5th) of *N. tabacum*, which contained comparatively lower accumulation of siRNAs and viral DNA than the non-recovered leaves (2nd and 3rd). This may be due to leaf/tissue-specific responses against the particular virus, in which various host factors might be actively involved. Previously, recovery of *N. benthamiana* carrying a functional RDR1 orthologue from *M. truncatula* against *Tomato ring spot virus* was observed (Jovel *et al.*, 2007). Similarly, *N. benthamiana* with truncated and non-functional RDR1 was found to result in the development of PVX infection symptoms (Yang *et al.*, 2004). These reports suggest that during ToLCGV infection, the recovery of leaves of *N. tabacum* possibly involved RDR1. However, ToLCNDV infection showed no recovery on *N. tabacum*, suggesting an inter-play between the host and viral components. RDR1 is essential for the generation and amplification of virus-specific siRNAs in plants infected with positive-stranded RNA viruses (Donaire *et al.*, 2008; Wang *et al.*, 2010); however, discrepancies in RDR1 function have been observed in various studies dependent upon the type of virus infection. For example, RDR1 in rice was essential to generate siRNAs in response to *Brome mosaic bromovirus* infection, but not for *Wheat dwarf geminivirus* (Chen *et al.*, 2010).

To identify the host factors involved in the RNA silencing process, expression of genes was analysed at different stages (recovered and non-recovered leaves) in both ToLCGV and ToLCNDV infections on *N. tabacum*. In contrast to ToLCNDV infection, genes associated with the TGS machinery (such as *AGO4*, *AGO7*, *DCL3*) were significantly down-regulated in leaves recovered from ToLCGV infection. The defense genes showed comparatively higher expression, especially in the tissues infected with ToLCNDV at 3 wpi, except for *RDR1*, which showed enhanced expression in ToLCGV-infected recovered tissue. The question arises as to why the activation of such genes failed to allow the plant to recover from infection? A possible explanation for this observation lies in the pattern of gene expression. We found that the RDR2-DCL3-AGO4-mediated methylation pathway genes were especially activated upon ToLCNDV infection, which targets the 24-nt siRNA-based methylation of the viral genome. Ideally, activation of this pathway should lead to the methylation of ToLCNDV; however, we observed that the level of cytosine methylation at the ToLCNDV intergenic region was significantly reduced. This suggests that the pathway is probably regulated at the translational level. On the other hand, the activation of RDR1 positively triggered the recovery of tissue from ToLCGV.

RDR polymerases (RDRPs) have been shown to have a vital role in recovery, and RDR6-deficient *N. benthamiana* plants lack any antiviral response (Xie *et al.*, 2001; Schwach *et al.*, 2005; Garcia-Ruiz *et al.*, 2010). It has been demonstrated that RDRPs play an integral role in the establishment of ToLCV infection in non-host Arabidopsis (Li *et al.*, 2014). RDR1 and RDR6 are the major RDRPs involved in both virus-specific small-RNA (vsRNA) biogenesis and antiviral silencing (Garcia-Ruiz *et al.*, 2010). In this study, *RDR1* was been found to maintain an enhanced level throughout ToLCGV infection. In contrast, the levels of *RDR6* and *RDR2* significantly increased in the 5th leaf (symptomatic) infected with ToLCNDV, whilst the level of *RDR1* remained significantly low. This also suggests possible cross-talk between RDR1 and RDR6 in *N. tabacum* plants inoculated with ToLCGV. RDR1 plays a dual role by either contributing in SA-mediated antiviral defense or by suppressing RDR6-mediated antiviral RNA silencing (Xie *et al.*, 2001; Ying *et al.*, 2010). RDR1 acts as a primary responder to viral RNA for a wide range of viruses while the function of RDR6 is limited to sensing viral RNAs once they reach a threshold concentration (Wang *et al.*, 2010). Hence, RDR6 plays an inconsequential role in the presence of RDR1. Low levels of RDR6 in recovered leaves can be linked to reduced accumulation of AGOs, DCLs, and RDR2. Therefore, the mechanism for triggering activation of the RDR1/RDR6-dependent pathway may be differentially regulated by viral proteins of DNA viruses (e.g. begomoviruses) in contrast to the RNA viruses.

These observations indicate the possible role of RNA silencing and the interplay between host and virus components during recovery from infection. This study showed that the dynamics of the host and virus counterparts, i.e. between the pre-coat protein (AV2) of the virus and the host’s RNA-silencing component RNA-dependent RNA polymerase 1 (NtRDR1), was crucial for providing symptom-remission attributes to *N. tabacum*. It is notable that recovery in *N. benthamiana* following ∆AV2 inoculation was positively correlated with elevated levels of siRNA. This observation is also supported by other studies where AV2 has been found to act as a major determinant of pathogenicity (Sharma and Ikegami, 2010). Further examination revealed that using a chimeric molecule (VANDAV2) to replace ToLCGV-AV2 with ToLCNDV-AV2 also impaired the symptom-remission process in *N. tabacum*. Moreover, significantly decreased levels of viral siRNAs were measured in systemic leaves of *N. tabacum*, in contrast to the corresponding recovered leaves inoculated with the wild-type virus. This was further substantiated by mutant inoculation on *N. benthamiana*. These species-specific responses may be correlated with the absence of functional RDR1 in *N. benthamiana*. The disappearance of symptoms had a significant correlation with the altered levels of viral AV2 (see Supplementary Fig. S12A). The decrease in the viral transcript levels over time following ∆AV2 inoculation can be explained as an inability of NbRDR1 to maintain the stability of the viral transcript after recovery (Supplementary Fig. S12B–D). As a result, the siRNA level becomes elevated due to increased activity of RDR6, which in turn is negatively regulated by RDR1 (Ying *et al.*, 2010). Hence, the blocking of symptom recovery by ToLCNDV-AV2 might be due to the differential reaction of host factor(s) specific to both TGS and PTGS, which may influence the recovery process.

Our study highlights a new finding whereby ToLCNDV-AV2 interferes with the RDR1-mediated antiviral pathway. Expression analysis of *NtRDR1* in leaves (L2 and L5) infiltrated with ToLCNDV and ToLCGV suggested that ToLCNDV-AV2 selectively blocked accumulation, while ToLCGV had limited effects on *NtRDR1* repression. The expression of RDR1 was found to be inversely proportional to the accumulation of *AV2* transcripts. Inoculation studies with the chimeric molecule (VANDAV2) and PVX-NAAV2 suggested that during the symptom-recovery process AV2 plays as a key role in maintaining the level of RDR1. A previous study has suggested that *N. benthamiana* has a truncated and non-functional RDR1 protein (Yang *et al.*, 2004). The infectivity of the wild-type and mutants of ToLCNDV on NbNtRDR1 plants implied that the ToLCNDV pre-coat protein can suppress the function of NtRDR1. However, inoculation of the NtRDR1-overexpression line of *N. benthamiana* with ToLCGV highlighted the inability of this virus to suppress the NtRDR1-mediated antiviral response. Recently, it was found that the down-regulation of *RDR1* expression in potato did not affect the resistance against PVX (Hunter *et al.*, 2016). In contrast, various previous studies had indicated that RDR1 was specifically involved in the defense against PVX and PVY in tobacco (Xie *et al.*, 2001; Rakhshandehroo *et al.*, 2009). Our results provide an explanation for this apparent inconsistency in RDR1 function. We may infer that RDR1 function and disease establishment depend upon the activity of a viral factor, which could efficiently suppress the host RNA-silencing mechanism.

Plants undergoing symptom recovery during the course of viral infection have a higher DNA methylation percentage of geminivirus genomes (Rodríguez-Negrete *et al.*, 2009; Sahu *et al.*, 2014). Our study showed that ToLCNDV had a relatively lower percentage of DNA methylation in the intergenic region (IR) region in comparison to ToLCGV-IR in the 5th leaf of *N. tabacum* at 3 wpi. Elevated levels of siRNAs have been linked with higher DNA methylation at the corresponding region of the geminivirus genome (Rodríguez-Negrete *et al.*, 2009; Sahu *et al.*, 2014). Even though RDR1 and/or RDR6 are involved in producing 21–22-nt siRNAs, a strong loss of DNA methylation has been reported in Arabidopsis mutants defective in RDR1/RDR6 (Nuthikattu *et al.*, 2013; Matzke and Matzke, 2014). Therefore, the observed low levels of DNA methylation might be the effect of reduced expression of RDR1 in ToLCNDV-infected *N. tabacum* plants. We used a NtRDR1-overexpression line of *N. benthamiana* to further evaluate the role of RDR1 in the DNA methylation, and found that the ToLCNDV genome showed no alteration in cytosine methylation upon NtRDR1 overexpression. This suggests that ToLCNDV can block the RDR1-mediated antiviral response, both in wild-type and transgenic *N. benthamiana*. Interestingly, overexpression of NtRDR1 resulted in 20% enhanced methylation within the ToLCGV genome. Recently, the role of RDR1 has been shown to be involved with the regulation of DNA methylation and abiotic stress responses in rice (Wang *et al.*, 2014*b*).

In conclusion, recovery from ToLCGV infection is specific to the host species, and is characterized by considerable and persistent decreases in both viral titer and symptom severity. The contrasting patterns of symptom development in tobacco plants inoculated with the two different begomoviruses clearly suggests that the establishment of disease symptoms in the host plant depends upon the interplay between the *AV2*-encoded pre-coat protein and RDR1. To the best of our knowledge, the present study provides the first report of ToLCNDV-AV2-regulated repression of NtRDR1-mediated antiviral silencing. In addition, RDR1 promotes methylation of the ToLCGV promoter, and contributes to the lower abundance of viral transcripts in transgenic plants and enhances levels of siRNAs. This could be a primary step towards understanding the natural responses of host plants to counter virus infections, and thus be exploited as a defense strategy against geminiviruses.

## Supplementary data

Supplementary data are available at *JXB* online.

Table S1. List of primers used in the present study.

Fig. S1. Phenotype of *N. tabacum* plants inoculated with ToLCNDV and ToLCGV.

Fig. S2. Relative accumulation of ToLCNDV-specific DNA and siRNAs.

Fig. S3. Relative levels of DNA-B accumulation of ToLCNDV and ToLCGV in *Nicotiana* spp. inoculated with either mutants or wild-type infectious clones.

Fig. S4. Relative levels of virus-specific siRNAs in *Nicotiana* spp.

Fig. S5. Relative levels of transcripts of various host factors of the RNAi machinery.

Fig. S6. Mapping of methylated cytosines along the length of ToLCNDV-IR in the leaves of wild-type *N. tabacum*.

Fig. S7. Mapping of methylated cytosines along the length of ToLCGV-IR in the leaves of wild-type *N. tabacum*.

Fig. S8. Mapping of methylated cytosines along the length of ToLCNDV-IR in the leaves of wild-type *N. benthamiana*.

Fig. S9. Mapping of methylated cytosines along the length of ToLCNDV-IR in the leaves of *N. benthamiana* lines overexpressing *NtRDR1*.

Fig. S10. Mapping of methylated cytosines along the length of ToLCGV-IR in the leaves of wild-type *N. benthamiana*.

Fig. S11. Mapping of methylated cytosines along the length of ToLCGV-IR in the leaves of *N. benthamiana* lines overexpressing *NtRDR1*.

Fig. S12. Relative levels of viral transcripts in *Nicotiana* spp. infected with wild-type and mutants of ToLCNDV.

Supplementary Material
