# Supplementary material for: Dynamics of a geminivirus-encoded pre-coat protein and host RNA-dependent RNA polymerase 1 in regulating symptom recovery in tobacco
Source: J Exp Bot. 2018 Feb 8;69(8):2085–102. doi: 10.1093/jxb/ery043 (PMC6019014; doi:10.1093/jxb/ery043)
Supplement: Supplementary Material [file ery043_suppl_suppl_table_figures.pdf]

Supplementary data

Table S1. List of primers used in the present study.

PRIMER		PRIMER SEQUENCE (5′-3′)
**Primers used for site directed mutagenesis of viral ORFs**		
NDVΔAC2	ΔAC2 FP	AATCAAGAATGCAGTCTTCATAACACTCGAAGAACCACTCTAT
	ΔAC2 RP	ATAGAGTGGTTCTTCGAGTGTTATGAAGACTGCATTCTTGATT
NDΔAC4	ΔAC4 FP	ATGGGTCTCCGCATATCCATGTAATCATCCAATTCGAAGGGAAATTC
	ΔAC4 RP	GAATTTCCCTTCGAATTGGATGATTACATGGATATGCGGAGACCCAT
NDΔAV2	ΔAV2 FP	GTTTGGATCCACAAACATGTGAGATCCATTATTGCACG
	ΔAV2 FP	CGTGCAATAATGGATCTCACATGTTTGTGGATCCAAAC
GVΔAC2	ΔAC2 FP	AAAATGCAACCTTCGTAACCCTCGAAGGCCCAC
	ΔAC2 RP	GTGGGCCTTCGAGGGTTACGAAGGTTGCATTTT
GVΔAC4	ΔAC4 FP	TGGGACTCCTCACTTGCATGTAATCATCCAGTTCGAAGGAAAG
	ΔAC4 RP	CTTTCCTTCGAACTGGATGATTACATGCAAGTGAGGAGTCCCA
GVΔAV2	ΔAV2 FP	AAGTATTGTAGTTCGTACTATGTGAGATCCTTTGTTAAACGAGTTTC
	ΔAV2 FP	GAAACTCGTTTAACAAAGGATCTCACATAGTACGAACTACAATACTT
VAAV2-	FP	GGCCCCAAGTATTGTAGTTACTAGTATGTGGGATCCTTTGTTAAACGAG
SpeI	RP	CTCGTTTAACAAAGGATCCCACATACTAGTAACTACAATACTTGGGGCC
VAAV2 –	FP	AGGAGCCCTGATGTGCCTAGGGGCTGTGAGGGTCCG
Avr II	RP	CGGACCCTCACAGCCCCTAGGCACATCAGGGCTCCT
NDAV2	FP	ACTAGTATGTGGGATCCATTATTGCACG
	RP	CCTAGGCACGTCGGGACTTCTATAC
**Primers used for quantitative Real Time PCR**		
**RDR1**	FP	AACAACAAAAGCGCGGAATT
	RP	TACTTCCCAACACTGCAAACTTCT
**AGO1**	FP	CCGCATCAGCCTGTACCATA
	RP	GGTTGTATTGCTTGGGTTGCA
**SGS3**	FP	AGATGTGGGTGAAATGAGTTATGAGA
	RP	GCCTGGACCACCTTTGCAT

**RDR6**	FP	AGTGGAGCCATTGGTAGGTGTAA
	RP	CTGACATGGACACCCCAAAAT
**AGO2**	FP	GCAAAGCCTAAAGCCTACATCTG
	RP	TCAAACCAACCAATGCATCCT
**DCL1**	FP	CTGGCCGATTAACCGACTTG
	RP	CATTTTTAACCTCACTCACGAAGTCA
**DCL2**	FP	GGGAAGTAGCGGCTTTGTCAT
	RP	CTACAAGCAGAGAAGGATCATGGAA
**DCL3**	FP	GAATGCGGTGAAGGCTCTGA
	RP	GAAGTGCAGCCGAATCTTGTG
**DCL4**	FP	TCATCAGTGGTTGCGTAAGAAAA
	RP	TCTTTACTTGTGATTCTTCGAAATCTG
**AGO4**	FP	TGGAGCACTCCCTCGAAATAA
	RP	CATCGGAATTTTTGCAGCAA
**RDR2**	FP	ATTGCCAGGCCCATTGAG
	RP	TCCCCCTTTGCATGACTAAC
**DRBP4**	FP	CCGCAACCTTCCTTCATCTG
	RP	GTGTCCACACTAGCCAATGCA
**AGO7**	FP	ACTCATCCGCACCCTCTTG
	RP	CTCCTGCCTATGCGTTTGG

Primers for DNA methylation study

**NDV_IR**	FP	ATGTGAGGAAATAGTTTTTGG
	RP	AATTTCATTGGTTGAGGG
**GV_IR**	NbFP1	CGTTAGAGAGCACTTGGGATAAGTG
	NbRP1	CTAACATACATCTAAAACCGTGAAC
	NtFP1	TTGGGATAAGTGAGAAAATAA
	NtRP1	ATTTATGCCATTGGAGTC
	NtFP2	TATGTATCGGTGTATTGGAGT
	NtRP2	GAACTACAATACTTGGGG

Primers used for cloning into pGR106 vector

ToLCGV-AC2 Cla1	FP	ATCGATATGCAACCTTCGTCACCCTC
ToLCGV-AC2 Not1	RP	GCGGCCGCTTAAATACCCTTAAGAAACGACCAG
ToLCNDV-AC2 Not1	FP	GCGGCCGCATGCAGTCTTCATCACACTCGAAGAACC

ToLCNDV-AC2 Sal1	RP	GTCGACTTAAGGACCTGGGTTTTGAAGACTCTC
ToLCGV-AV2 Cla1	FP	ATCGATATGTGGGATCCTTTGTTAAACGAG
ToLCGV-AV2 Not1	RP	GCGGCCGCTCAGGGCTCCTGTACATTCTGTAG
ToLCNDV-AV2 Cla1	FP	ATCGAT ATGTGGGATCCATTATTGC
ToLCNDV-AV2 Not1	RP	GCGGCCGCCTATACATTCTGTACATTCTGGG

Legends of Supplementary Figures

Figure S1. Phenotype of the *N. tabacum* plant inoculated with tomato infecting begomoviruses.

Top view of phenotypes corresponding to mock, ToLCNDV and ToLCGV-infected *N. tabacum* plants (A) Mock (B) ToLCNDV and (C) ToLCGV infected plants. (D)1st, 2nd,3rd and 4th leaves showing progressive recovery on *N. tabacum* infected with ToLCGV. (E-H) *N. tabacum* infected with ToLCGV at 1, 2, 3 and 4 wpi. Leaves are labeled as 1st, 2nd, 3rd and 4th from the first symptomatic leaf onwards. (I) Symptom on recovered tobacco cv. Xanthi following re-inoculation with ToLCGV (left) and ToLCNDV (right) at 8 wpi. .

Figure S2. Relative accumulation of ToLCNDV-specific DNA and siRNAs.

(A) Sampling pattern of *N. tabacum* leaves. (B) Viral DNA accumulation of ToLCNDV in *N. tabacum* leaves (1st to 5th) at 21 dpi (3 wpi). (C) ToLCNDV-specific siRNA accumulation in *N. tabacum* leaves (1st to 5th) at 3 wpi. Enriched small RNA and miR-160 blots are shown at the bottom serve as the loading control.

Figure S3. Relative level of DNA-B accumulation of ToLCNDV and ToLCGV in *Nicotiana* spp. inoculated with either mutants or wild type infectious clones.

Accumulation of DNA-B of ToLCNDV (A), and ToLCGV (B) in *N. benthamiana* inoculated with either mutants or wild type infectious clones. Accumulation of DNA-B of ToLCNDV (C) and ToLCGV (D) in *N. tabacum* inoculated with either mutants or wild type infectious clones. Ethidium bromide stained total genomic DNA at the bottom serves as the loading control. Viral replicative forms are shown: OC, open circular; Lin, linear; SS, single stranded; SC, super coiled.

Figure S4. Relative level of virus-specific siRNAs in *Nicotiana* spp.

Virus specific-siRNA accumulation in *N. benthamiana* (A) and *N. tabacum* (B) plants inoculated with mutants and wild type ToLCNDV at 3 wpi. (C) Virus specific-siRNA accumulation in *N. benthamiana* inoculated with mutants and wild type ToLCGV at 3 wpi. (D-E) Comparison of virus- derived siRNA accumulation in *N. benthamiana* (D), *N. tabacum* (E) inoculated with mutants and wild type ToLCNDV at 1, 2, 3 and 4 wpi. (F) Relative accumulation of virus-specific siRNA in *N. benthamiana* plants inoculated with mutants and wild type ToLCGV at 3 wpi. siRNA blots were hybridized with [α-^{32}P]-dCTP labeled

overlapping region of ORF AC1- AC2 and AC2-AC3. Ethidium bromide stained enriched small RNAs and miR-160 blots placed at the bottom serves as the loading control. X-axis represents wpi where as Y-axis represents relative (%) siRNA accumulation.

Figure S5. Relative level of transcripts of various host factors of RNAi machinery.

Bar graph represent comparative accumulation of AGO1 (A), AGO2 (B), AGO4 (C), AGO7 (D), SGS3 (E), DRBP (F), DCL1 (G), DCL2 (H), DCL3 (I) and DCL4 (J) on the 2nd and 5th leaves of *N. tabacum* inoculated with both ToLCNDV and ToLCGV at 9dpi and 21dpi. Actin gene was used as a reference gene for the normalization of relative abundances. The values represent means of three biological replicates.

Figure S6. Mapping of methylated cytosines along the length of ToLCNDV-IRin the leaves of wild-type *N. tabacum*.

(A) Position of cytosines methylation in bisulfite-treated DNA corresponding to ToLCNDVIR region. (B) Alignment of bisulfite sequenced clones. Methylated cytosines are highlighted with green font and non-methylated cytosines by red font. A total of 5 clones from bisulfite treated samples were sequenced and analyzed.

Figure S7. Mapping of methylated cytosines along the length of ToLCGV-IR in the leaves of wild-type *N. tabacum*.

(A) Target region of ToLCGV-IR and primer synthesis, (B) Figure represents level of methylated cytosines in ToLCGV-IR (part-1 and part-2). Methylated cytosines are highlighted with green font and non-methylated cytosines by red font. A minimum of five clones from each corresponding fragments were sequenced from bisulfite treated samples.

Figure S8. Mapping of methylated cytosines along the length of ToLCNDV-IR in the leaves of wild-type *N. benthamiana*.

(A) Position of cytosines methylation in bisulfite-treated DNA corresponding to ToLCNDV-IR region. (B) Alignment of bisulfite sequenced clones. Methylated cytosines are highlighted with green font and non-methylated cytosines by red font. A minimum of five clones were from bisulfite treated samples were sequenced and analyzed.

Figure S9. Mapping of methylated cytosines along the length of ToLCNDV-IR in the leaves of *N. benthamiana* lines overexpressing *NtRDR1*.

(A) Position of cytosines methylation in bisulfite-treated DNA corresponding to ToLCNDV-IR region. (B) Alignment of bisulfite sequenced clones. Methylated cytosines are highlighted with green font and non-methylated cytosines by red font. A minimum of five clones were from bisulfite treated samples were sequenced and analyzed.

Figure S10. Mapping of methylated cytosines along the length of ToLCGV-IR in the leaves of wild-type *N. benthamiana*.

(A) Position of cytosines methylation in bisulfite-treated DNA corresponding to ToLCGV-IR region. (B) Alignment of bisulfite sequenced clones. Methylated cytosines are highlighted with green font and non-methylated cytosines by red font. A minimum of five clones were from bisulfite treated samples were sequenced and analyzed.

Figure S11. Mapping of methylated cytosines along the length of ToLCGV-IR in the leaves of *N. benthamiana* lines overexpressing *NtRDR1*.

(A) Position of cytosines methylation in bisulfite-treated DNA corresponding to ToLCGV-IR region. (B) Alignment of bisulfite sequenced clones. Methylated cytosines are highlighted with green font and non-methylated cytosines by red font. A minimum of five clones were from bisulfite treated samples were sequenced and analyzed.

Figure S 12. Relative level of viral transcripts in *Nicotiana* spp. infected with wild-type and mutants of ToLCNDV.

(A) Detection of *AV2* transcripts in *N. tabacum* plants inoculated with either NA+NB or VA+VB at 3 wpi. Detection of viral transcript in *N. benthamiana* plants at 1 wpi (B), 2 wpi (C) and 3 wpi (D). [α-^{32}P] dCTP labeled AC1 of ToLCNDV was used as probe. Ethidium bromide stained rRNA at the bottom serves as the loading control. Symbol * represents the significance at P<0.001 and ** denotes P<0.003.

Figure S1

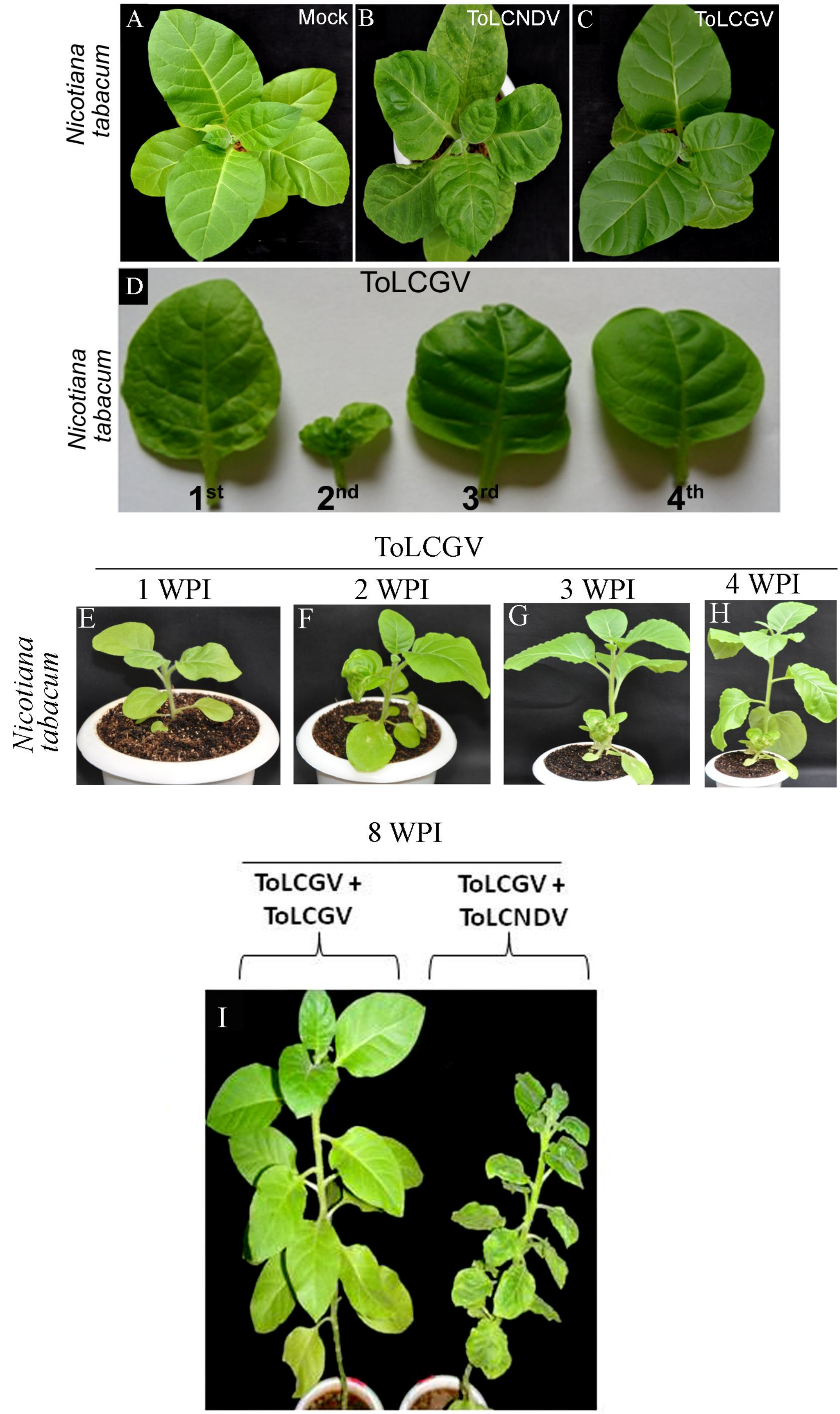

Nicotiana tabacum
A
Mock
B
ToLCNDV
C
ToLCGV
Nicotiana tabacum
D
ToLCGV
1st
2nd
3rd
4th
ToLCGV
1 WPI
2 WPI
3 WPI
4 WPI
Nicotiana tabacum
E
F
G
H
8 WPI
ToLCGV + ToLCGV
ToLCGV + ToLCNDV
I

Figure S2

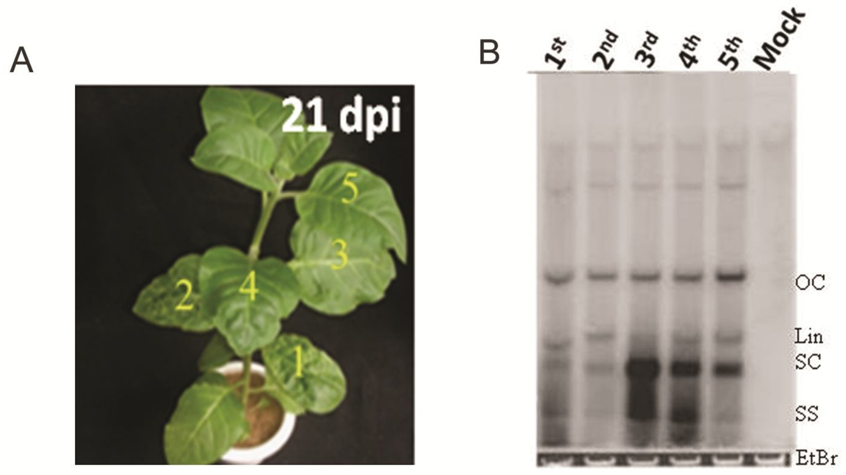
A
21 dpi
1
2
3
4
5
B
1st
2nd
3rd
4th
5th
Mock
OC
Lin
SC
SS
EtBr
C
Mock
L1
L2
L3
L4
L5
24 nt
21 nt
miR-160
Enriched small RNAs

Figure S3

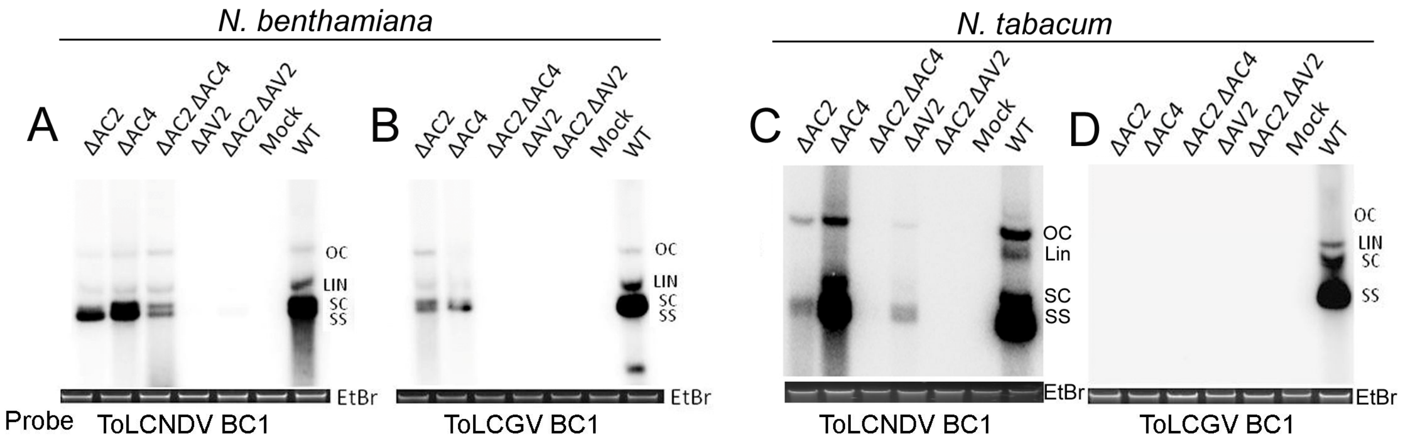
N. benthamiana
N. tabacum
A
ΔAC2
ΔAC4
ΔAC2 ΔAC4
ΔAV2
ΔAC2 ΔAV2
Mock
WT
OC
LIN
SC
SS
EtBr
Probe
ToLCNDV BC1
B
ToLCGV BC1
C
Lin
D

Figure S4

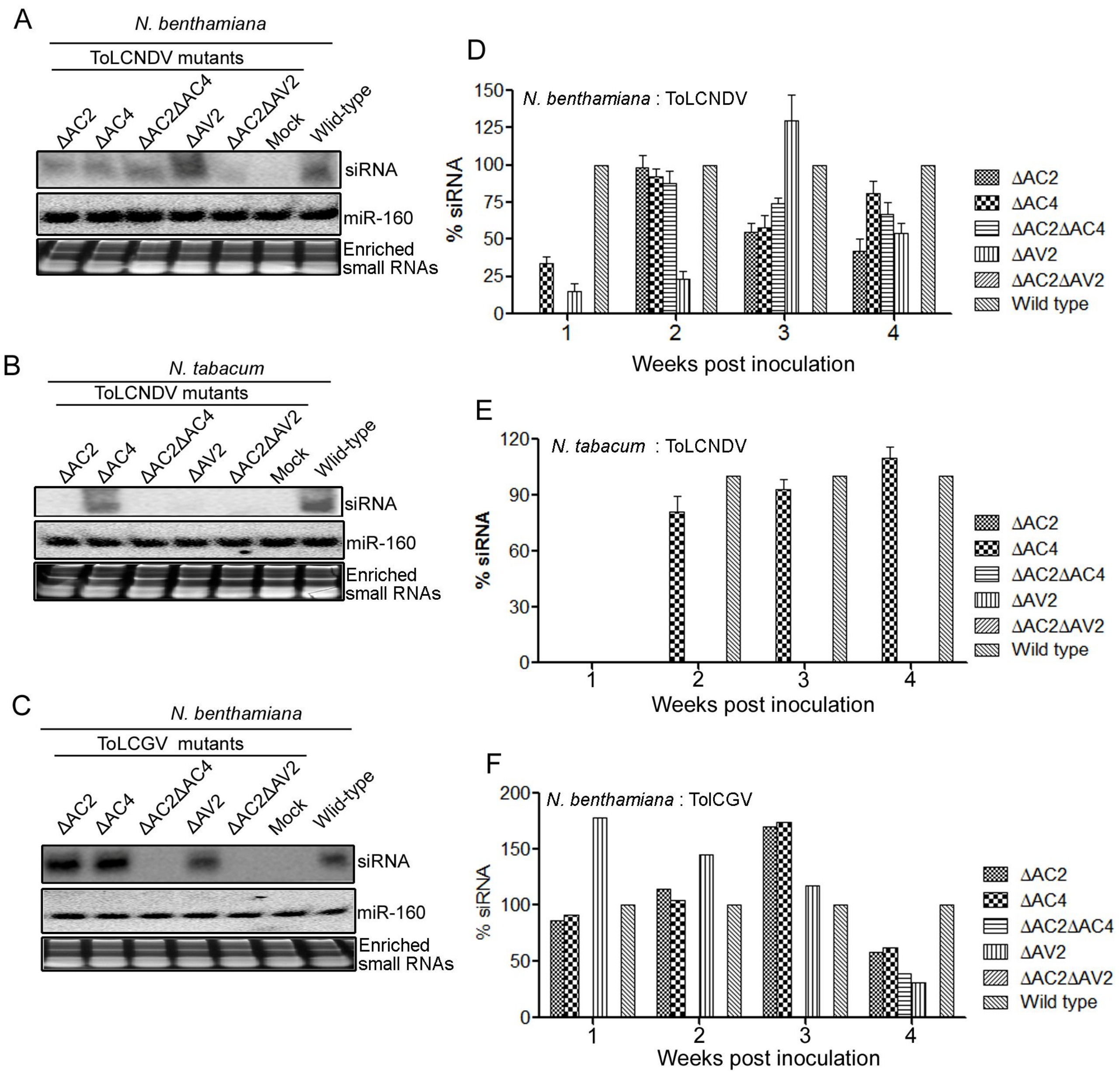
A
N. benthamiana
ToLCNDV mutants
ΔAC2
ΔAC4
ΔAC2ΔAC4
ΔAV2
ΔAC2ΔAV2
Mock
Wlid-type
siRNA
miR-160
Enriched small RNAs
B
N. tabacum
ToLCNDV mutants
ΔAC2
ΔAC4
ΔAC2ΔAC4
ΔAV2
ΔAC2ΔAV2
Mock
Wlid-type
siRNA
miR-160
Enriched small RNAs
C
N. benthamiana
ToLCGV mutants
ΔAC2
ΔAC4
ΔAC2ΔAC4
ΔAV2
ΔAC2ΔAV2
Mock
Wlid-type
siRNA
miR-160
Enriched small RNAs
D
N. benthamiana : ToLCNDV
% siRNA
150
125
100
75
50
25
0
1
2
3
4
Weeks post inoculation
ΔAC2
ΔAC4
ΔAC2ΔAC4
ΔAV2
ΔAC2ΔAV2
Wild type
E
N. tabacum : ToLCNDV
% siRNA
120
90
60
30
0
1
2
3
4
Weeks post inoculation
ΔAC2
ΔAC4
ΔAC2ΔAC4
ΔAV2
ΔAC2ΔAV2
Wild type
F
N. benthamiana : TolCGV
% siRNA
200
150
100
50
0
1
2
3
4
Weeks post inoculation
ΔAC2
ΔAC4
ΔAC2ΔAC4
ΔAV2
ΔAC2ΔAV2
Wild type

Figure S5

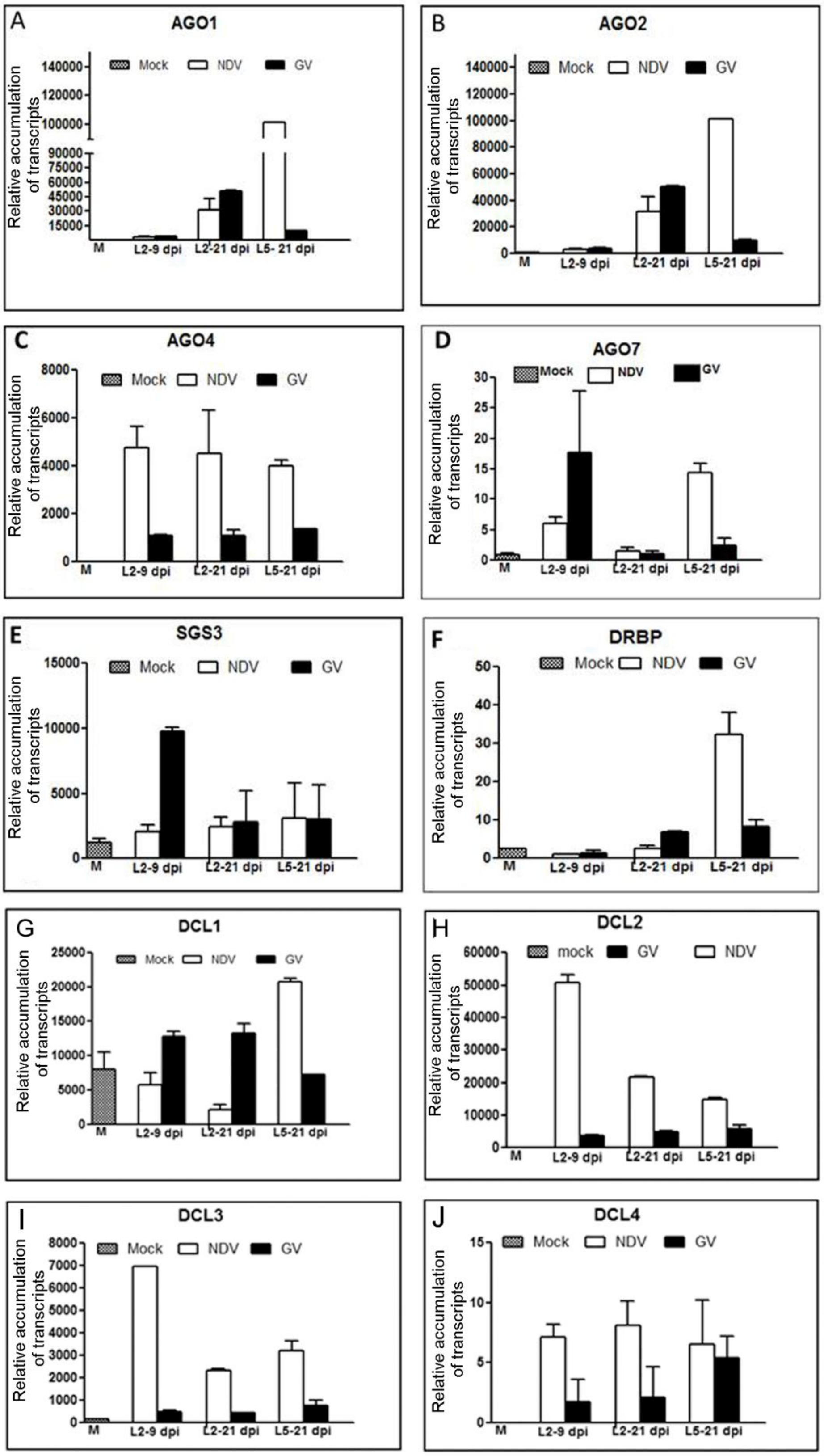

A
AGO1
B
AGO2
C
AGO4
D
AGO7
E
SGS3
F
DRBP
G
DCL1
H
DCL2
I
DCL3
J
DCL4
Mock
NDV
GV
mock
Relative accumulation of transcripts
M
L2-9 dpi
L2-21 dpi
L5-21 dpi
L5- 21 dpi

Figure S6

A

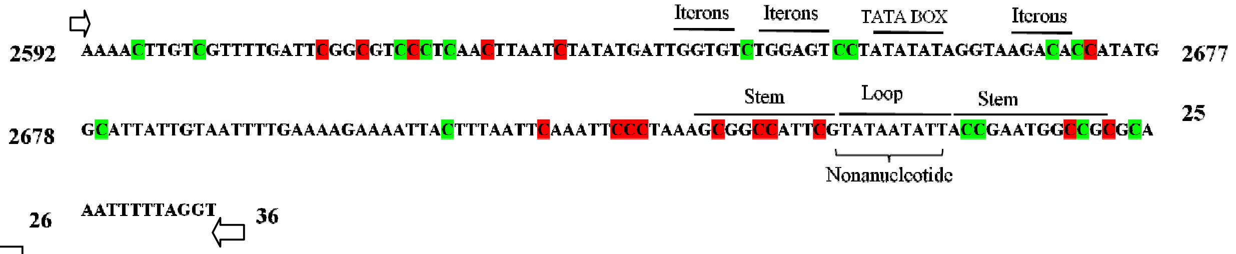

B

```
D10              AAAACTTGTCGTTTTGATTTGGTGTCTCTCAACTTAATCTATATGATTGGTGTCTGGAGT 60
E10              AAAACTTGTCGTTTTGATTTGGTGTCTCTCAACTTAATCTATATGATTGGTGTCTGGAGT 60
B11              AAAACTTGTCGTTTTGATTTGGTGTCTCTCAACTTAATCTATATGATTGGTGTCTGGAGT 60
B4               AAAACTTGTCGTTTTGATTCGGCGTCCCTCAATTTAATCTATATGATTGGTGTCTGGAGT 60
A4               AAAACTTGTCGTTTTGATTCGGCGTCCCTCAACTTAATTTATATGATTGGTGTCTGGAGT 60
IR               AAAACTTGTCGTTTTGATTCGGCGTCCCTCAACTTAATCTATATGATTGGTGTCTGGAGT 60

D10              CCTATATATAGGTAAGACACCATATGGCATTATTGTAATTTTGAAAAGAAAATTACTTTA 120
E10              CCTATATATAGGTAAGACACCATATGGCATTATTGTAATTTTGAAAAGAAAATTACTTTA 120
B11              CCTATATATAGGTAAGACACCATATGGCATTATTGTAATTTTGAAAAGAAAATTACTTTA 120
B4               CCTATATATAGGTAAGACACTAAATGGCATTATTGTAATTTTGAAAAGAAAATTACTTTA 120
A4               CCTATATATAGGTAAGACACTAAATGGCATAATTGTAATTTTGAAAAGAAAATTACTTTA 120
IR               CCTATATATAGGTAAGACACCATATGGCATTATTGTAATTTTGAAAAGAAAATTACTTTA 120

D10              ATTCAAATTCCCTAAAGCGGCCATTCGTATAATATTACCGAATGGTCGCGCAAATTTTTA 180
E10              ATTCAAATTCCCTAAAGCGGCCATTCGTATAATATTACCGAATGGTCGCGCAAATTTTTA 180
B11              ATTCAAATTTTTTAAAGTGGTTATTTGTATAATATTACCGAATGGCCGCGCAAATTTTTA 180
B4               ATTCAAATTCCTTATAGCGGCCATTCGTATAATATTACCGAATGGCCGCGCAAATTTTTA 180
A4               ATTTAAATTCCTTATAGCGGTCATTTGTATAATATTACCGAATGGCCGTGCAAATTTTTA 180
IR               ATTCAAATTCCCTAAAGCGGCCATTCGTATAATATTACCGAATGGCCGCGCAAATTTTTA 180

D10              GGT
E10              GGT
B11              GGT
B4               GGT
IR               GGT
                 ***
```

Figure S7

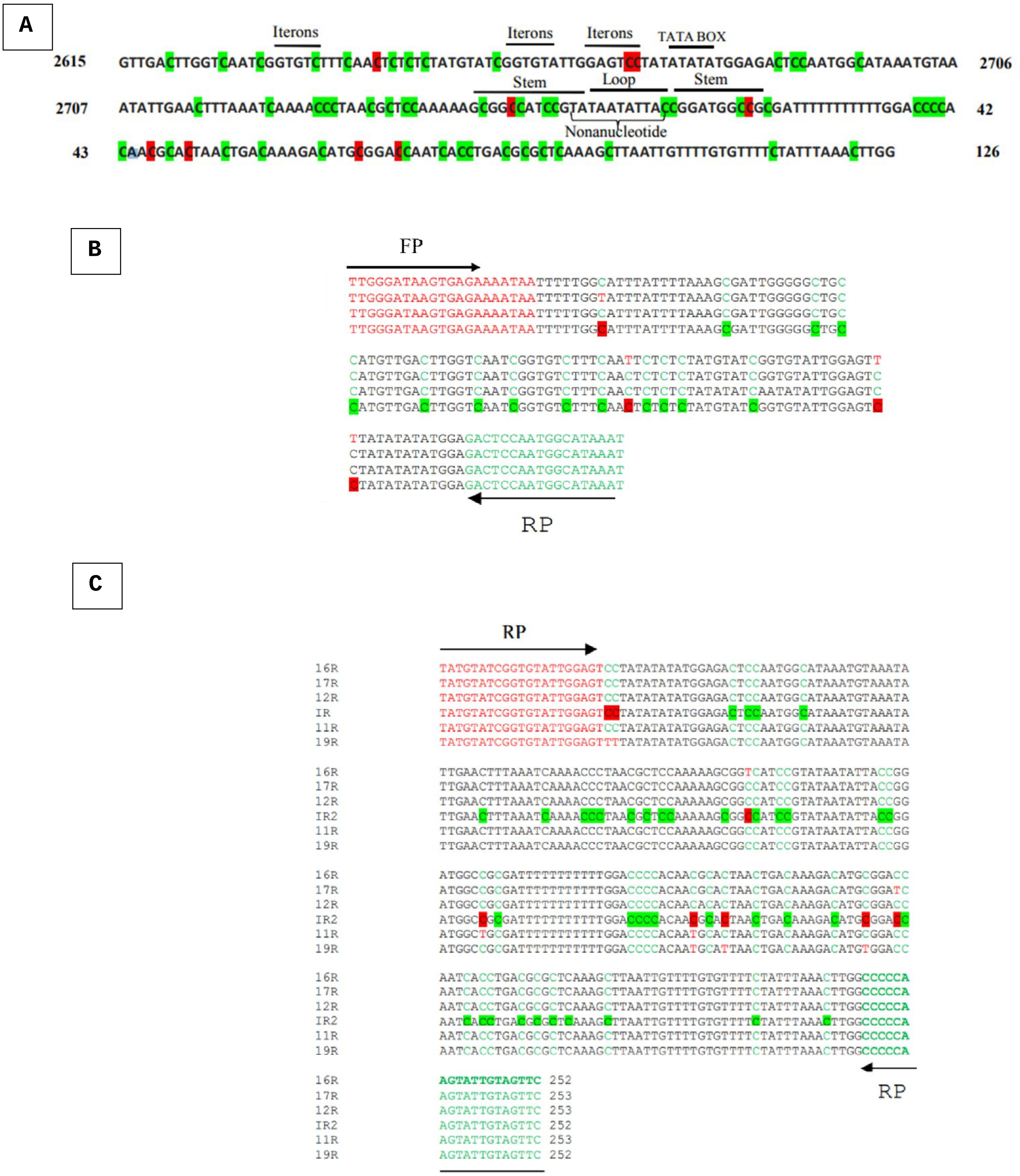
A
Iterons
Iterons
Iterons
TATA BOX
Stem
Loop
Stem
Nonanucleotide
2615
2706
2707
42
43
126
B
FP
RP
C
RP
16R
17R
12R
IR
IR2
11R
19R
252
253
RP

Figure S8

A

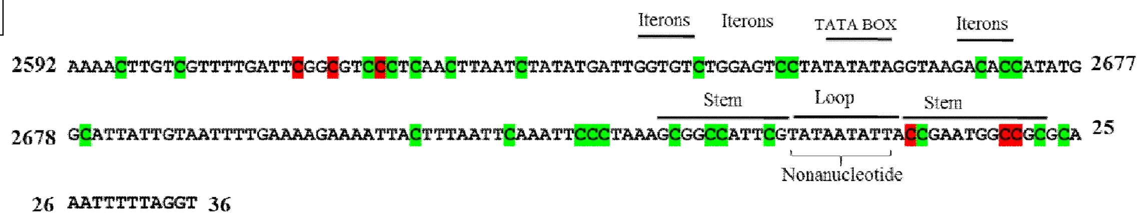

B

```
NWT42           AAAACTTGTCGTTTTGATTTGGTGTCTCTCAACTTAATCTATGTGATTGGTGGCTGGAGT
NWT47           AAAACTTGTCGTTTTGATTTGGTGTCTCTCAACTTAATCTATGTGATTGGTGTCTGGAGT
NWT43           AAAACTTGTCGTTTTGATTTAGTGTCTCTCAACTTAATCTATATGATTGGTGTCTGGAGT
NWT45           AAAACTTGTCGTTTTGATTTGGTGTCTCTCAACTTAATCTATGTGATTGGTGTCTGGAGT
NWT48           AAAACTTGTCGTTTTGATTTGGTGTCTCTCAACTTAATCTATGTGATTGGTGTCTGGAGT
NWT44           AAAACTTGTCGTTTTGATTTGGTGTCTCTCAACTTAATCTATATGATTGGTGTCTGGAGT
NDVIR           AAAACTTGTCGTTTTGATTCGGCGTCCCTCAACTTAATCTATATGATTGGTGTCTGGAGT

NWT42           CCTATATATAGGTAAGACACCATATGGCATTATTGTAATTTTGAAAAGAAAATTACTTTA
NWT47           CCTATATATAGGTAAGACACCATATGGCATTATTGTAATTTTGAAAAGAAAATTACTTTA
NWT43           CCTATATATAGGTAAGACACCATATGGCATTATTGTAATTTTGAAAAGAAAATTACTTTA
NWT45           CCTATATATAGGTAAGACACCATATAGCATTATTGTAATTTTGAAAAGAAAATTACTTTA
NWT48           CCTATATATAGGTAAGACACCATATAGCATTATTGTAATTTTGAAAAGAAAATTACTTTA
NWT44           CCTATATATAGGTAAGACACCATATGGCATTATTGTAATTTTGAAAAGAAAATTACTTTA
NDVIR           CCTATATATAGGTAAGACACCATATGGCATTATTGTAATTTTGAAAAGAAAATTACTTTA

NWT42           ATTCAAATTCCCTAAAGCGGCCATTCGTATAATATTATCGAATGGTTGCGCAAATTTTTA
NWT47           ATTCAAATTCCCTAAAGCGGCCATTCGTATAATATTACCGAATGGCCGCGCAAATTTTTA
NWT43           ATTCAAATTCCCTAAAGCGGCCATTCGTATAATATTACCAAATGGCCGCGCAAATTTTTA
NWT45           ATTCAAATTCCCTAAAGCGGCCATTCGTATAATATTACCAAATGGCCGCGCAAATTTTTA
NWT48           ATTCAAATTCCCTAAAGCGGCCATTCGTATAATATTACCGAATGGCCGCGCAAATTTTTA
NWT44           ATTCAAATTCCCTAAAGCGGCCATTCGTATAATATTACCGAATGGCCGCGCAAATTTTTA
NDVIR           ATTCAAATTCCCTAAAGCGGCCATTCGTATAATATTACCGAATGGCCGCGCAAATTTTTA

NWT42           GGT
NWT47           GGT
NWT43           GGT
NWT45           GGT
NWT48           GGT
NWT44           GGT
NDVIR           GGT
```

Figure S9

A

```
                                                                 Iterons  Iterons    TATA BOX          Iterons
2592 AAAACTTGTCGTTTTGATTCGGCGTCCCTCAACTTAATCTATATGATTGGTGTCTGGAGTCCTATATATAGGTAAGACACCATATG 2677
                                                                  Stem        Loop        Stem
2678 GCATTATTGTAATTTTGAAAAGAAAATTACTTTAATTCAAATTCCCTAAAGCGGCCATTCGTATAATATTACCGAATGGCCGCGCA 25
                                                                     Nonanucleotide
26 AATTTTTAGGT 36
```

B

```
NRD54          AAAACTTGTCGTTTTGATTTGGTGTCTCTCAACTTTATCTATGTAATTGGTGTCTGGAGT
NRD51          AAAACTTGTCGTTTTGATTTGGTGTCTCTCAACTTTATCTATGTAATTGGTGTCTGGAGT
NRD53          AAAACTTGTCGTTTTGATTTGGTGTCTCTCAACTTTATCTATGTAATTGGTGTCTGGAGT
NRD50          AAAACTTGTCATTTTAATTTGGTGTCTCTCAACTTTATCTATGTAATCGGTGTCTGGAGT
NRD55          AAAACTTGTCGTTTTGATTTGGTGTCTCTCAACTTTATCTATGTAATTGATGTCTGGAGT
NRD52          AAAACTTGTCGTTTTGATTTGGTGTCTCTCAACTTTATCTATGTAATTGGTGTCTGGAGT
NDVIR          AAAACTTGTCGTTTTGATTCGGCGTCCCTCAACTTAATCTATATGATTGGTGTCTGGAGT

NRD54          CCTATATATAGGTAAGACACCATATGGCATTATTGTAATTTTGAAAAGAAAATTACTTTA
NRD51          CCTATATATAGGTAAGACACCATATGGCATTATTGTAATTTTGAAAAGAAAATTACTTTA
NRD53          CCTATATATAGGTAAGACACCATATGGCATTATTGTAATTTTGAAAAGAAAATTACTTTA
NRD50          CCTATATATAGGTAAGACACCATATGGCATTATTGTAATTTTGAAAAGAAAATTACTTTA
NRD55          CCTATATATAGGTAAGACACCATATGGCATTATTGTAATTTTGAAAAGAAAATTACTTTA
NRD52          CCTATATATAGGTAAGACACCATATGGCATTATTGTAATTTTGAAAAGAAAATTACTTTA
NDVIR          CCTATATATAGGTAAGACACCATATGGCATTATTGTAATTTTGAAAAGAAAATTACTTTA

NRD54          ATTCAAATTCCCTAAAGCGGCCATTCGTATAATATTACCGAATGGTTGTGCAAATTTTTA
NRD51          ATTCAAATTCCCTAAAGCGGCCATTCGTATAATATTACCGAATGGCCGCGCAAATTTTTA
NRD53          ATTCAAATTCCCTAAAGCGGCCATTCGTATAATATTACCGAATGGCCGCGCAAATTTTTA
NRD50          ATTCAAATTCCCTAAAGCGGCCATTCGTATAATATTACCGAATGGCCGCGCAAATTTTTA
NRD55          ATTCAAATTCCCTAAAGCGGCCATTCGTATAATATTACCGAATGGCCGCGCAAATTTTTA
NRD52          ATTCAAATTCCCTAAAGCGGCCATTCGTATAATATTACCGAATGACCGCGCAAATTTTTA
NDVIR          ATTCAAATTCCCTAAAGCGGCCATTCGTATAATATTACCGAATGGCCGCGCAAATTTTTA

NRD49          GGT
NRD54          GGT
NRD51          GGT
NRD53          GGT
NRD50          GGT
NRD55          GGT
NRD52          GGT
NDVIR          GGT
```

Figure S10

A

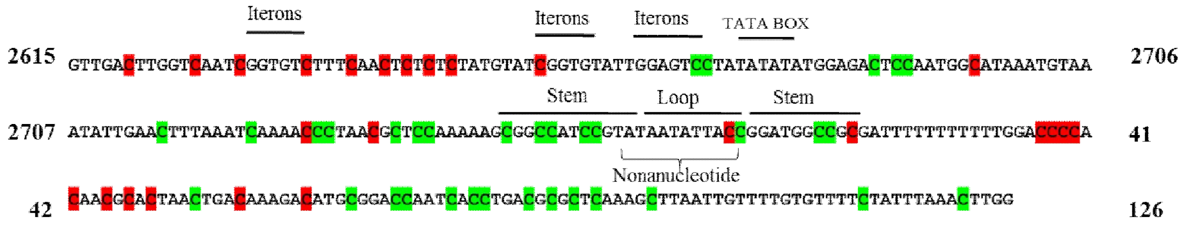

B

```
GWT63          GTTGACTTGGTCAATCGGTGTCTTTCAACTCTCTCTATGTATCGGTGTATTGGAGTCCTA
GWT58          GTTGACTTGGTCAATTGGTGTTTTTTAATTTTTTTATGTATTGGTGTATTGGAGTCCTA
GWT60          GTTGACTTGGTCAATCGGTGTCTTTCAACTCTCTCTATGTATCGGTGTATTGGAGTCCTA
GWT61          GTTGATTTGGTCAATTGGTGTCTTTCAACTCTCTCTATGTATCGGTGTATTGGAGTCCTA
GWT62          GTTGACTTGGTCAATCGGTGTCTTTCAACTCTCTCTATGTATCGGTGTATTGGAGTCCTA
GWT59          GTTGATTTGGTTAATTGGTGTCTTTCAACTCTCTCTATGTATCGGTGTATTGGAGTCCTA
IRGV           GTTGACTTGGTCAATCGGTGTCTTTCAACTCTCTCTATGTATCGGTGTATTGGAGTCCTA

GWT63          TATATATGGAGACTCCAATGGTATGAATGTAAATATTGAACTTTAAATCAAAACCCTAAC
GWT58          TATATATGGAGACTCCAATGGCATAAATGTAAATATTGAACTTTAAATCAAAACCCTAAC
GWT60          TATATATGGAGACTCCAATGGCATAAATGTAAATATTGAACTTTAAATCAAAACCCTAAC
GWT61          TATATATGGAGACTCCAATGGCATAAATGTAAATATTGAACTTTAAATCAAAATCCTAAC
GWT62          TATATATGGAGACTCCAATGGCATAAATGTAAATATTGAACTTTAAATCAAAACCCTAAC
GWT59          TATATATGGAGACTCCAATGGCATAAATGTAAATATTGAACTTTAAATCAAAACCCTAAT
IRGV           TATATATGGAGACTCCAATGGCATAAATGTAAATATTGAACTTTAAATCAAAACCCTAAC

GWT63          GCTCCAAAAAGCGGCCATCCGTATAATATTACCGGATGGCCGCGATTTTTTTTTTTGGAC
GWT58          GCTCCAAAAAGCGGCCATCCGTATAATATTACCGGATGGCCGCGATTTTTTTTTTTGGAC
GWT60          GCTCCAAAAAGCGGCCATCCGTATAATATTACCGGATGGCCGCGATTTTTTTTTTTGGAC
GWT61          GCTCCAAAAAGCGGCCATCCGTATAATATTACCGGATGGCCGCGATTTTTTTTTTTGGAC
GWT62          GCTCCAAAAAGCGGCCATCCGTATAATATTACCGGATGGCCGTGATTTTTTTTTTTGGAT
GWT59          GCTCCAAAAAGCGGCCATCCGTATAATATTATCGGATGGCCGCGATTTTTTTTTTTGGAT
IRGV           GCTCCAAAAAGCGGCCATCCGTATAATATTACCGGATGGCCGCGATTTTTTTTTTTGGAC

GWT63          CCCACAACGCACTAACTGACAAAGACATGCGGACCAATCACCTGACGCGCTCAAAGCTTA
GWT58          CCCACAACGCATTAACTGACAAAGACATGCGGACCAATCACCTGACGCGCTCAAAGCTTA
GWT60          CCCACAACGCACTAACTGACAAAGACATGCGGACCAATCACCTGACGCGCTCAAAGCTTA
GWT61          CCCACAACGCACTAACTGACAAAGACATGCGGACCAATCACCTGACGCGCTCAAAGCTTA
GWT62          TTTATAATGTACTAACTGACAAAGACATGCGGACCAATCACCTGACGCGCTCAAAGCTTA
GWT59          TTTACAACGCACTAACTGATAAAGATATGCGGACCAATCACCTGACGCGCTCAAAGCTTA
IRGV           CCCACAACGCACTAACTGACAAAGACATGCGGACCAATCACCTGACGCGCTCAAAGCTTA

GWT63          ATTGTTTTGTGTTTTCTATTTAAACTTGG
GWT58          ATTGTTTTGTGTTTTCTATTTAAACTTGG
GWT60          ATTGTTTTGTGTTTTCTATTTAAACTTGG
GWT61          ATTGTTTTGTGTTTTCTATTTAAACTTGG
GWT62          ATTGTTTTGTGTTTTCTATTTAAACTTGG
GWT59          ATTGTTTTGTGTTTTCTATTTAAACTTGG
IRGV           ATTGTTTTGTGTTTTCTATTTAAACTTGG
```

Figure S11

A

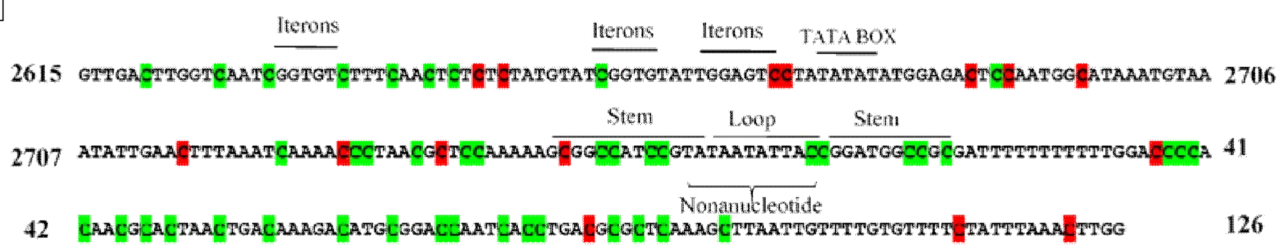

B

```
GRD64          GTTGACTTGGTCAATCGGTGTCTTTCAACTCTCTCTATGTATCGGTGTATTGGAGTTTTA
GRD65          GTTGACTTGGTCAATCGGTGTCTTTCAACTCTCTCTATGTATCGGTGTATTGGAGTTTTA
GRD68          GTTGACTTGGTCAATCGGTGTCTTTCAACTCTCTCTATGTATCGGTGTATTGGAGTCCTA
GRD69          GTTGACTTGGTCAATCGGTGTCTTTCAACTCTTTTTATGTATCGGTGTATTGGAGTCCTA
GRD70          GTTGACTTGGTCAATCGGTGTCTTTCAACTCTTTTTATGTATCGGTGTATTGGAGTCCTA
IRGV           GTTGACTTGGTCAATCGGTGTCTTTCAACTCTCTCTATGTATCGGTGTATTGGAGTCCTA

GRD64          TATATATGGAGATTCTAATGGTATAAATGTAAATATTGAACTTTAAATCAAAATCCTAAC
GRD65          TATATATGGAGACTCCAATGGCATAAATGTAAATATTGAATTTTAAATCAAAACCCTAAC
GRD68          TATATATGGAGACTCCAATGGCATAAATGTAAATATTGAATTTTAAATCAAAACCCTAAC
GRD69          TATATATGGAGACTCCAATGGCATAAATGTAAATATTGAACTTTAAATCAAAACCCTAAC
GRD70          TATATATGGAGACTCCAATGGCATAAATGTAAATATTGAACTTTAAATCAAAACCCTAAC
IRGV           TATATATGGAGACTCCAATGGCATAAATGTAAATATTGAACTTTAAATCAAAACCCTAAC

GRD64          GCTCCAAAAAGCGGCCATCCGTATAATATTACCGGATGGCCGCGATTTTTTTTTTTTGGAC
GRD65          GCTCCAAAAAGCGGCCATCCGTATAATATTACCGGATGGCCGCGATTTTTTTTTTTTGGAC
GRD68          GTTCCAAAAAGTGGCCATCCGTATAATATTACCGGATGGCCGCGATTTTTTTTTTTTGGAC
GRD69          GCTCCAAAAAGCGGCCATCCGTATAATATTACCGGATGGCCGCGATTTTTTTTTTTTGGAT
GRD70          GTTCCAAAAAGTGGCCATCCGTATAATATTACCGGATGGCCGCGATTTTTTTTTTTTGGAC
IRGV           GCTCCAAAAAGCGGCCATCCGTATAATATTACCGGATGGCCGCGATTTTTTTTTTTTGGAC

GRD64          CCCACAACGCACTAACTGACAAAGACATGCGGACCAATCACCTGATGCGCTCAAAGCTTA
GRD65          CCCACAACGCACTAACTGACAAAGACATGCGGACCAATCACCTGATGCGCTCAAAGCTTA
GRD68          CCCACAACGCACTAACTGACAAAGACATGCGGACCAATCACCTGACGCGCTCAAAGCTTA
GRD69          CCCACAACGCACTAACTGACAAAGACATGCGGACCAATCACCTGACGCGCTCAAAGCTTA
GRD70          CCCACAACGCACTAACTGACAAAGACATGCGGACCAATCACCTGACGCGCTCAAAGCTTA
IRGV           CCCACAACGCACTAACTGACAAAGACATGCGGACCAATCACCTGACGCGCTCAAAGCTTA

GRD64          ATTGTTTTGTGTTTTCTATTTAAACTTGG
GRD65          ATTGTTTTGTGTTTTCTATTTAAACTTGG
GRD68          ATTGTTTTGTGTTTTTTATTTAAATTTGG
GRD69           ATTGTTTTGTGTTTTCTATTTAAACTTGG
GRD70           ATTGTTTTGTGTTTTCTATTTAAACTTGG
IRGV           ATTGTTTTGTGTTTTCTATTTAAACTTGG
```

Figure S12

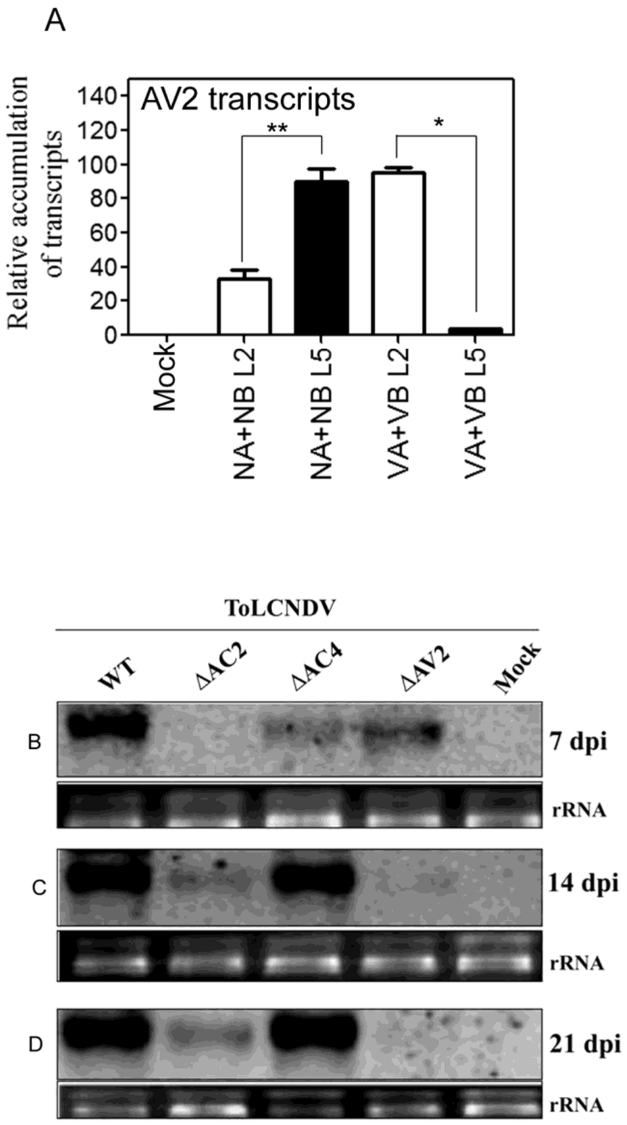
A
AV2 transcripts
Relative accumulation of transcripts
140
120
100
80
60
40
20
0
**
*
Mock
NA+NB L2
NA+NB L5
VA+VB L2
VA+VB L5
ToLCNDV
WT
ΔAC2
ΔAC4
ΔAV2
Mock
B
7 dpi
rRNA
C
14 dpi
rRNA
D
21 dpi
rRNA
